# Quantitative analysis of redox proteome reveals oxidation-sensitive protein thiols acting in fundamental processes of developmental hematopoiesis

**DOI:** 10.1016/j.redox.2022.102343

**Published:** 2022-05-23

**Authors:** K. Pimkova, M. Jassinskaja, R. Munita, M. Ciesla, N. Guzzi, P. Cao Thi Ngoc, M. Vajrychova, E. Johansson, C. Bellodi, J. Hansson

**Affiliations:** a Lund Stem Cell Center, Division of Molecular Hematology, Lund University, Lund, Sweden; b BIOCEV, 1st Medical Faculty, Charles University, Vestec, Czech Republic; c Biomedical Research Center, University Hospital Hradec Kralove, Hradec Kralove, Czech Republic

**Keywords:** Redox proteomics, Hematopoiesis, Leukemia, Developmental biology, Cysteine oxidative modifications, Protein translation, HSPCs, hematopoietic stem and progenitor cells, MLL-ENL, mixed-lineage leukemia-eleven-nineteen leukemia, Cys, cysteine

## Abstract

Fetal and adult hematopoietic stem and progenitor cells (HSPCs) are characterized by distinct redox homeostasis that may influence their differential cellular behavior in normal and malignant hematopoiesis. In this work, we have applied a quantitative mass spectrometry-based redox proteomic approach to comprehensively describe reversible cysteine modifications in primary mouse fetal and adult HSPCs. We defined the redox state of 4,438 cysteines in fetal and adult HSPCs and demonstrated a higher susceptibility to oxidation of protein thiols in fetal HSPCs. Our data identified ontogenic changes to oxidation state of thiols in proteins with a pronounced role in metabolism and protein homeostasis. Additional redox proteomic analysis identified oxidation changes to thiols acting in mitochondrial respiration as well as protein homeostasis to be triggered during onset of MLL-ENL leukemogenesis in fetal HSPCs. Our data has demonstrated that redox signaling contributes to the regulation of fundamental processes of developmental hematopoiesis and has pinpointed potential targetable redox-sensitive proteins in *in utero*-initiated MLL-rearranged leukemia.

## 1 Introduction

Hematopoietic stem and progenitor cells (HSPCs) are located atop the hematopoietic hierarchy and include stem cells as well as early multipotent progenitors which give rise to all mature blood cells [1]. Along development, the process of blood cell development (hematopoiesis) occurs in waves with distinct functional characteristics between fetal and adult HSPCs [[2], [3], [4]]. The focus of fetal hematopoiesis is to supply the growing embryo with blood cells and to produce sufficient stem cells for adult life [5]. This major wave of maturation and expansion of HSPCs occurs in the fetal liver (FL) and has its largest burst at embryonic day 14.5 in mice [6,7]. Before birth, HSPCs begin to populate the bone marrow, where hematopoiesis is maintained after birth and for the rest of mammalian life [4]. Consistent with the demand for rapid expansion of the blood cell pool in the embryo, FL HSPCs are actively proliferating and have a higher translation rate as well as higher metabolic demands than their adult bone marrow (ABM) counterparts [2,8,9]. For this reason, FL HSPCs are believed to preferentially employ efficient oxygen-dependent generation of energy, e.g. mitochondrial oxidative phosphorylation, which results in increased levels of reactive oxygen species (ROS) [10].

Low or moderate levels of ROS in fetal HSPCs are essential for signaling processes controlling proliferation, differentiation and self-renewal [11]. However, accumulation of ROS as a consequence of e.g. genetic alterations, or exposure to extrinsic sources, can lead to oxidative damage and become harmful for the cell [12,13], possibly even causing cancer [14]. To avoid cellular damage, ROS levels must be tightly controlled by cellular antioxidant defense systems. The primary sensors of elevated ROS are protein cysteine thiols. Depending on the levels of ROS and the local protein cysteine environment, protein thiols can be covalently reversibly or irreversibly modified. While the reversible cysteine post-translational modifications (PTMs) modulate protein function and act as oxidation-reduction (redox) switches in cellular signaling, modifications considered to be irreversible (to sulfinic or sulfonic acid) may lead to protein denaturation and severe protein damage [15]. Our recent comprehensive proteomic comparison of fetal and adult HSPCs revealed that the fetal cells have significantly lower levels of antioxidant enzymes, including those that employ thiols for elimination of ROS, such as the thioredoxin and glutathione/glutaredoxin systems [2]. Considering the elevated oxidative state in fetal relative to adult HSPCs [10], these findings suggest that fetal HSPCs have a distinct redox homeostasis and are more sensitive to changes in the redox environment compared to adult HSPCs.

Although there is clear evidence for a role of ROS signaling in hematopoiesis [16,17], the protein targets of redox modifications regulating normal and malignant blood cell development remain to be identified. Comprehensive identification and site-specific quantification of protein cysteine modifications under steady state conditions is essential to fully understand the role of redox regulation in HSPCs. Mass spectrometry (MS)-based redox proteomics represents a powerful analytical tool for studying redox signaling [18]. However, due to the low abundance of cysteines in the proteome, current redox proteomics protocols require large amounts of starting material [[19], [20], [21], [22], [23], [24]]. This represents a major technical challenge for the analysis of redox signaling in HSPCs, which are rare, relatively small in size, and, until very recently, have been difficult to expand *in vitro* [25].

In this study, we have applied a sequential iodoTMT labelling strategy and a nanoLC-MS^3^ method to characterize the redox state of cysteines in 400,000 fluorescence-activated cell sorting (FACS)-purified primary fetal and adult HSPCs. In agreement with the divergent nature of fetal and adult hematopoiesis, we show that the redox molecular landscape is distinct between fetal and adult HSPCs, and forms an important component of HSPC regulation along ontogeny. We demonstrate that an increased oxidative state in fetal HSPCs is essential for redox signaling in these cells and reveal redox-sensitive cysteines acting in fundamental functional properties of HSPCs during ontogeny and leukemia development.

## 2 Results

### 2.1 Quantitative proteomics identifies protein targets of redox modification in fetal and adult blood stem and progenitor cells

We first investigated the relationship between intracellular redox state, free thiols and cell cycle state in fetal and adult HSPCs (defined as Lineage^−^ Sca-1^+^ c-Kit^+^ [LSK]) and found that the cells with high or intermediate oxidative state (Ox^int/high^) represent a substantial proportion of all fetal HSPC subpopulations, including long-term hematopoietic stem cells (LT-HSCs) which reside almost exclusively in a Ox^low^ state in the adult [26,27] (SF1).

To identify which protein thiols are targets of redox modification in early hematopoietic cells along ontogeny, we subjected FACS-purified LSK HSPCs from mouse FL and ABM in three replicates (SF2a) to an in-depth MS-based quantitative and site-specific proteomic analysis (Fig. 1a). We applied sequential labelling of free and selectively reduced protein thiols by taking advantage of iodoTMT reagents; a set of six isobaric isomers that are iodoacetyl-activated and covalently and irreversibly label sulfhydryl (-SH) groups of reduced cysteines in proteins, which can subsequently be captured through affinity enrichment (SF2b-c). Using this approach on a starting material of 400,000 sorted cells (corresponding to approximately 20 μg of protein) per sample, we identified a total of 4,164 unique cysteine peptides (4,438 unique cysteine [Cys] sites) corresponding to 1,850 unique protein groups (Fig. 1b, Supplementary Table 1). Of these, 3,376 and 3,357 peptides were quantified with valid values in all three replicates of fetal HSPCs and adult HSPCs, respectively. Our optimized conditions resulted in a cysteine enrichment efficiency of over 87% for each replicate (SF2c, d). The measured peptide oxidation level (SF2e) was reproducible between biological replicates (SF2g), with an average Pearson correlation coefficient of 0.864 and 0.792 in fetal and adult HSPCs, respectively (SF2f).

**Fig. 1:**
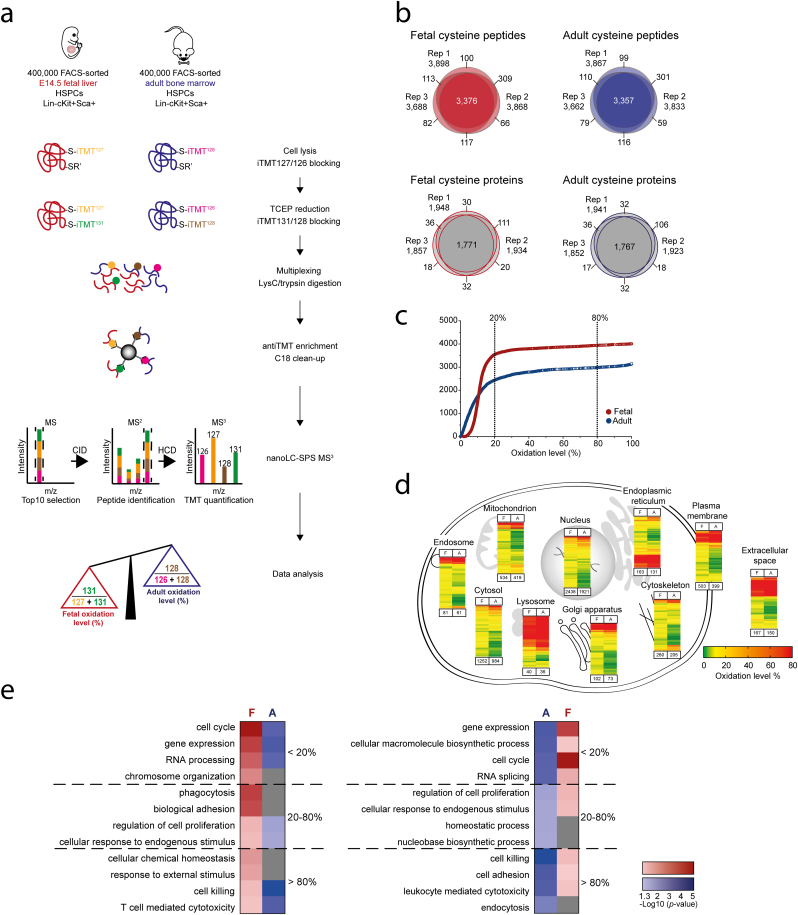
**Quantitative proteomics identifies redox-sensitive cysteines in fetal and adult HSPCs.** **a** Workflow for quantitative and site-specific detection of redox state of protein thiols in fetal and adult HSPCs. 400,000 FACS sorted Lin^−^ Sca-1^+^ cKit^+^ (LSK) hematopoietic stem and progenitor cells (HSPCs) per replicate from fetal liver E14.5 and adult bone marrow were processed using a redox proteomic workflow with sequential iodoTMT labelling, separated by nano liquid chromatography (nanoLC) and analyzed by mass spectrometry (MS) using synchronous precursor selection MS^3^ method (SPS-MS^3^). Raw intensities of each reporter were used to calculate the oxidation level of each peptide by generating a ratio of reversibly oxidized thiols and total available cysteines of the peptide. **b** The number and overlap of unique cysteine peptides and unique cysteine proteins with valid quantitative value either for free or oxidized thiol in three biological replicates identified by MS in fetal and adult HSPCs. **c** Cumulative distribution of oxidation level within the fetal and adult HSPC redoxome. Vertical lines show the marks for classification into three oxidation level groups (<20%, 20–80% and >80%). **d** Subcellular localization assigned to peptides identified in fetal (F) and adult (A) HSPCs, based on GO slim terms. Heatmaps represent the oxidation level (%) of unique peptides identified in fetal and adult HSPCs. The number of identified peptides is indicated below heatmaps. **e** The four most significantly enriched (p-value < 0.05) GO biological processes for each class of peptide oxidation level in fetal (F; left) and adult (A; right) HSPCs and their overlap with their adult or fetal counterparts, respectively, are displayed. Processes that were not detected are depicted in grey.

The 4,438 identified cysteine sites were mapped to 493 previously described reversibly modified sites, including intra-disulphide, S-nitrosylation and sulfenic acid modifications, as well as to 42 described disulphide bonds retrieved from UniProt (Supplementary Table 1). The oxidation level of identified peptides spanned the entire spectrum of 0–100% in both fetal and adult HSPCs (Fig. 1c, Supplementary Table 1). We classified all quantified peptides into three groups based on the peptide oxidation level (0–20%, 20–80%, 80–100%; Fig. 1c). Peptides with the highest oxidation level were preferentially assigned to lysosomes, extracellular space and endoplasmic reticulum (ER), while the majority of cytoplasmic and nuclear peptides were oxidized less than 20% (Fig. 1d). Gene ontology (GO) enrichment analysis showed that proteins with at least one cysteine residue oxidized at levels below 20% were significantly enriched for cell cycle and gene expression in both fetal and adult HSPCs (Fig. 1e). While fetal proteins with cysteines oxidized at a level of 20–80% were enriched for phagocytosis, endocytosis-related proteins were enriched in the group of proteins that contained cysteines with the highest oxidation level (>80%) in adult cells. In both fetal and adult HPSCs, proteins related to T-cell mediated cytotoxicity, as well as cell killing, were found in the group of proteins where cysteine oxidation was higher than 80% (Fig. 1e), indicating an especially high oxidation state of cysteines in cytotoxic proteins. Collectively, these data point to similar spatial distribution of protein redox regulation in fetal and adult HPSCs.

### 2.2 Fetal cysteine proteins in HSPCs show a higher degree of redox modulation than adult cysteine proteins

In contrast to adult HSPCs, where the majority of peptides were oxidized to less than 9%, our data showed that the majority of peptides in fetal HSPCs carried a cysteine oxidation level corresponding to 9–16% (Figs. 1c and 2a, and SF3a). This finding is in accordance with the higher oxidative state in fetal compared to adult HSPCs (SF1), as well as the lower level of antioxidant enzymes and higher proliferation and metabolic activity of fetal relative to adult cells [2,10]. Interestingly, adult HSPCs instead showed a higher percentage of peptides with high oxidation level (>70%), resulting in a slightly higher average oxidation level compared to fetal HSPCs (Fig. 2a). In line with this, cysteines known to form disulphides showed a median oxidation level of 78% and 95% for fetal and adult HSPCs, respectively (Supplementary Table 1), suggesting that adult proteins are more prone to form disulphides. We found 227 Cys sites with significantly different (adjusted p-value < 0.05) state of their thiol residue (reduced (-SH) and oxidized (-Sox) form) evaluated as an oxidation level between fetal and adult HSPCs (Fig. 2b). These belonged to 205 peptides (from 180 unique proteins). The majority of these (174 peptides belonging to 153 unique proteins) were higher oxidized in fetal HSPCs, while 31 peptides (belonging to 27 unique proteins) were higher oxidized in adult HSPCs. The median oxidation level for all 205 differentially oxidized peptides was 11% in fetus and 3% in adult (Fig. 2c). This suggests that redox switches and oxidative modifications with regulatory functions are different from disulphide bonds, and are acting at physiological levels of cellular cysteine oxidation.

**Fig. 2:**
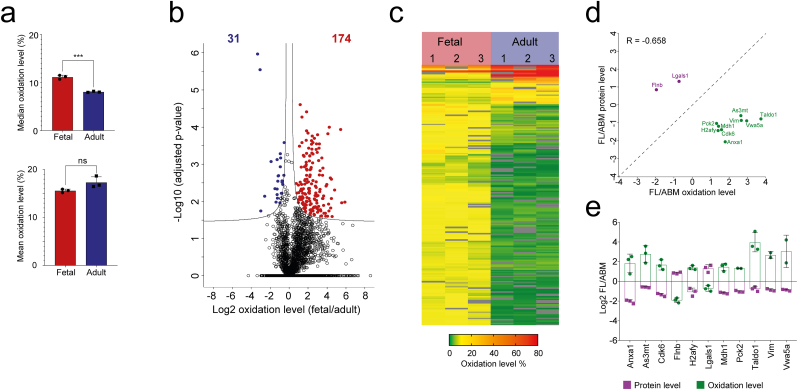
**Fetal cysteine proteins in HSPCs show a higher degree of redox modulation.** **a** Median (upper) and mean (lower) oxidation level for all identified and quantified peptides in fetal and adult HSPCs. Bars display mean ± SD from three biological replicates. **b** Volcano plot displaying average log2(fetal/adult) % oxidation for 4,164 unique cysteine containing peptides plotted against −log10 adjusted p-value. Unique cysteine containing peptides with significantly (adjusted p-value < 0.05) increased oxidation levels in fetal and adult HSPCs are highlighted in red and blue, respectively. The number of significantly differentially oxidized peptides are depicted in red (higher oxidized in fetal) and blue (higher oxidized in adult), respectively. **c** Heatmap representing oxidation level (%) of significantly differentially oxidized peptides (adjusted p-value < 0.05) in three biological replicates (1, 2, 3) of fetal and adult HSPCs. **d** Scatter plot depicting correlation of peptide oxidation changes and protein expression changes for significantly differentially oxidized cysteines between fetal (FL) and adult (ABM) HSPCs and significantly different protein expression detected by Jassinskaja et al. [2] in fetal and adult HSPCs. Pearson correlation coefficient is indicated. **e** Log2 fold change of peptides with significantly changed oxidation level (fetal/adult) (green) and proteins with significantly changed expression level (fetal/adult) (purple), respectively, are represented by the bar plot. ***p < 0.001 and ns = non-significant. (For interpretation of the references to color in this figure legend, the reader is referred to the Web version of this article.)

We mapped the peptide oxidation data to the corresponding protein expression data described by us previously [2] (Supplementary Tables 1 and 3). While we did not observe a significant overall correlation between peptide level changes and changes to their oxidative state (Pearson correlation, R = −0.0663; SF3b), we observed an interesting inverse relationship between strong changes to cysteine oxidation and strong changes to the expression level of the corresponding protein (Fig. 2, Fig. 3e). For example, the protein Igf2bp3, which is almost exclusively expressed during fetal hematopoiesis [2], was detected with extremely high thiol oxidation (100%) at two sites in adult HSPCs (Supplementary Table 1). This finding excludes the possibility that increased oxidation level is caused by increased protein expression alone. Furthermore, one of the proteins with opposite expression and cysteine oxidation change (H2afy; Fig. 3e) was found to undergo irreversible cysteine modification in a separate experiment (data not shown) suggesting that proteins prone to thiol oxidation may acquire higher oxidative states leading to irreversible oxidation, protein damage and subsequent proteasomal degradation [28].

**Fig. 3:**
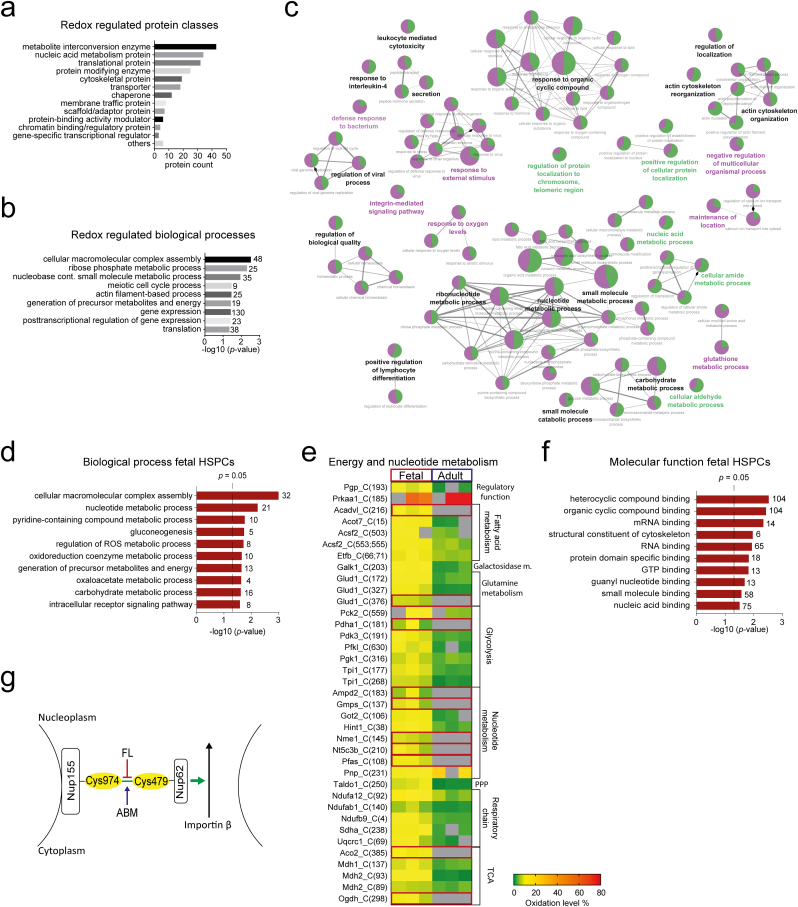
**Redox modulation of HSPC proteins forms an additional layer of regulation in fetal cells.** **a-b** PANTHER protein classes (a) and GO enrichment of biological processes (b) of redox regulated proteins (with significantly differentially oxidized cysteine(s) between fetal and adult HSPCs, and/or oxidation on the specific cysteine site(s) only in fetus or adult cells). **c** Functionally grouped annotation network (analyzed by ClueGO) [29] of two protein clusters: protein level differences (purple) and protein oxidation differences (green; if more peptides of one protein were identified the peptide with the highest oxidation is included) between fetal and adult HSPCs. The size of the node reflects the statistical significance of the term for both clusters. The relative proportion of the respective color in the pie chart is equal to the relative proportion of genes from each cluster associated with the enriched GO term. The degree of connectivity between terms (edges) is calculated using kappa statistics. The group leading term is the most significant term of the group. The label color of the group leading term represents the cluster (protein level or protein oxidation) with majority of genes that are enriched for the terms belonging to the group. Common background was used as a reference set. **d** GO enrichment for proteins with cysteines significantly higher oxidized in fetal HSPCs for overrepresentation of biological processes. Redundant GO terms were filtered out. **e** Heatmap represents oxidation level (%) of peptides with significantly changed cysteine oxidation (adjusted p-value < 0.05) between fetal and adult HSPCs and peptides with cysteines found to be oxidized only in fetus (3: 0 fetal: adult valid values; marked with red frames) that are involved in metabolic processes in three biological replicates. TCA: Tricarboxylic acid cycle; PPP: Pentoso-phosphate pathway; m.: metabolism. **f** GO enrichment for proteins with cysteines significantly higher oxidized in fetal HSPCs for overrepresentation of molecular function. Redundant GO terms were filtered out. **g** Schematic picture of cysteine oxidation of nuclear pore complex proteins, which affects macromolecular transport between the cytoplasm and the nucleoplasm. (For interpretation of the references to color in this figure legend, the reader is referred to the Web version of this article.)

### 2.3 Redox modulation of HSPC proteins forms a regulatory component of metabolic adaptation and protein homeostasis in fetal cells

We further investigated the characteristics of the redox regulated proteins. This group contains i) proteins with oxidation on the specific cysteine site only in fetal or adult cells, and ii) proteins with significantly differentially oxidized cysteine between fetal and adult HSPCs (Supplementary Table 3). The protein classes of these 279 proteins included transporters, chaperones, protein modifying enzymes, and protein-binding activity modulators (Fig. 3a). GO enrichment analysis revealed that cysteine oxidation in HSPCs controls ribose phosphate metabolic process, meiotic cell cycle process and translation (Fig. 3b). Most of these processes are regulated also by protein expression levels (Fig. 3c), emphasizing crosstalk between redox signaling and regulation by protein level. Interestingly, some processes appeared to be preferentially regulated by oxidative modification, including protein localization and metabolism of nucleic acids, while glutathione metabolic process and response to oxygen levels show a stronger regulation at the level of protein expression (Fig. 3c). We found 33 redox regulated proteins involved in RNA metabolism and pre-mRNA splicing and processing (SF4a). Among them 10 proteins have RNA helicase domain whose cysteines are conserved among species and Cys571 of Ddx1 is conserved among RNA helicases Ddx6 and Ddx18 (SF4b, SF8a, b). ‘DNA/RNA helicase, DEAD/DEAH box type, N-terminal’ domain was found as an overrepresented domain among redox regulated proteins (SF4c).

Proteins with significantly higher oxidized cysteines in adult HSPCs were enriched for processes related to proliferation and lymphocyte differentiation (including Lgals1 and Lgals9; SF4d). Proteins with significantly higher oxidized cysteines in fetal HSPCs were instead enriched for metabolic processes, such as ‘gluconeogenesis’, ‘regulation of reactive oxygen species metabolic process’ and ‘nucleotide metabolic process’ (Fig. 3d and e). 6 catalyzing enzymes of glycolysis and 4 proteins of the tricarboxylic acid (TCA) cycle showed significantly higher oxidation in fetal HSPCs or oxidation only in fetal HSPCs (Fig. 3e). Interestingly, we identified several components of the mitochondrial respiratory chain to be significantly higher oxidized in fetal HSPCs, including three non-catalytic subunits of respiratory complex I (Ndufab1, Ndufa12, Ndufb9), and catalytic subunits of complex II (Sdha) and complex III (Uqcrc1) (SF4e). We also found Cys185 of the AMP-protein kinase Prkaa1, a key redox and metabolic sensor [30], to be almost two times higher oxidized in adult relative to fetal HSPCs (on average 90% compared to 49% in fetal HSPCs) (Fig. 3e). Activation of the AMP kinase switches on catabolic processes while switching off anabolic ones [31]. Overall, the observed oxidation state of metabolic proteins is reminiscent of the described key role of cysteine oxidation in the metabolic adaptation to pro-oxidative environments [21,31,32].

We identified 23 proteins involved in protein quality control mechanisms to be differentially redox regulated between fetal and adult HSPCs (SF4f). This included 4 subunits of ATP dependent molecular chaperone complex (chaperonin-containing T-complex (TRiC)), 3 peptides of Heat shock protein HSP 90-alpha, 5 components of 26S proteasome complex and 2 ubiquitin conjugating enzymes. Among the 4 Cys sites on Hsp90aa1 that we found with increased oxidation in fetal HSPCs (SF4f), modification (S-nitrosylation) of Cys599 has been demonstrated to negatively regulate nitric oxide synthase (SF4g) [33], providing a feedback mechanism for limiting production of nitric oxide and thus possibly regulating mobilization and proliferation of HSPCs [34]. We found the highly conserved Cys273 of the ubiquitin conjugating enzyme (Uba1) oxidized only in fetal HSPCs (SF4f). Oxidation of this cysteine causes a conformational change that strongly decreases the first step in ubiquitin conjugation that marks cellular proteins for degradation through the ubiquitin-proteasome system (SF4h) [35]. Interestingly, in addition to 5 components of the 26S proteasome being differentially oxidized between fetal and adult HSPCs, we found Psmg1, a chaperone protein which promotes assembly of the 20S proteasome as part of a heterodimer with Psmg2, significantly higher oxidized in adult HSPCs. Furthermore, Psmb8, which we have found strongly higher expressed in adult HSPCs together with the other components of the immunoproteasome [2], showed oxidation uniquely in fetal HSPCs, again in line with our observation of an inverse correlation between strong expression and oxidation differences between fetal and adult HSPCs (Fig. 2d and e). All together, these findings suggest that altered protein degradation in fetal HSPCs is regulated by protein oxidation on top of protein expression differences in the proteasome system.

Intriguingly, various types of binding, including ‘mRNA binding’ and ‘GTP binding’, were overrepresented among molecular functions enriched in proteins significantly higher oxidized in fetal HSPCs (Fig. 3f), pointing to a redox-regulated ontogenic effect on molecular interactions. A large group of redox regulated proteins were involved in ’cellular macromolecular complex assembly’ (Fig. 3d). Within one of the largest protein complexes in the cell, the nuclear pore complex, which mediates macromolecular transport between the cytoplasm and the nucleoplasm, we identified 3 subunits (Nup88, Nup155 and Nup160) to be redox regulated. Interestingly, Nup155-Cys974, which was significantly higher oxidized in ABM HSPCs, has been shown to form a disulphide bond with Nup62-Cys479, which was almost 5 times higher oxidized in adult cells (quantified in one replicate, Supplementary Table 1, Fig. 3g), and this crosslink positively affect nuclear influx of importin beta cargo proteins [36,37]. The largest subgroup of proteins within the redox-regulated enriched process ‘cellular macromolecular complex assembly’ were a group of 7 translation initiation factors. Translation was also among the most overrepresented biological processes (Fig. 3b). This group additionally involved 2 re-initiation factors, 2 elongation factors and 8 ribosomal proteins. Similar to the reveal of thiol sites of several components of the translation machinery in yeast being sensitive to redox regulation [19], our data point to a probable role of redox signaling in the regulation of translation in HSPCs.

### 2.4 Oxidation of 43S pre-initiation complex proteins is under regulatory control in HSPCs

Detailed analysis of differentially oxidized translation initiation factors and ribosomal proteins revealed several components of the 43S pre-initiation complex (PIC) with a strongly altered protein oxidation status in fetal relative to adult HSPCs (Fig. 4a). This included a differential cysteine oxidation of at least one peptide of each protein subunit of 43S PIC with the exception of eukaryotic initiation factor (eIF) 2, suggesting a role of protein thiol oxidation in canonical cap-dependent translation initiation in fetal HSPCs (Fig. 4a and SF5a). Intriguingly, while most of the proteins of 43S PIC with differentially oxidized cysteines showed higher oxidation in fetal HSPCs, eIF3D and Rps11 showed higher oxidation level of Cys437 and 438, and Cys60, respectively, in adult HSPCs (Fig. 4, Fig. 5b). This suggests that the redox state of protein cysteines belonging to the 43S PIC do not solely reflect the higher oxidative environment in fetal HSPCs, but that their oxidation is under regulatory control in fetal and adult HSPCs.

**Fig. 4:**
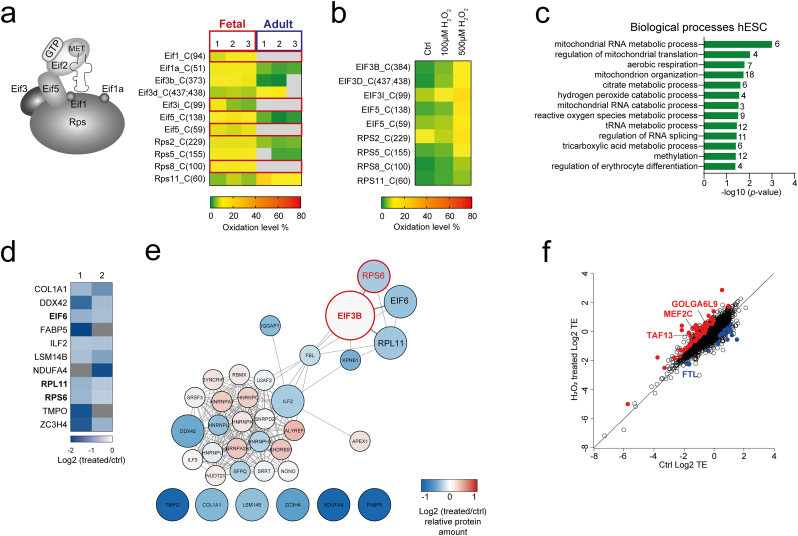
**Translation machinery components are sensitive to redox changes in mouse hematopoietic and human embryonic stem cells.** **a** Composition of the 43S translation PIC. Heatmap represents oxidation level (%) of significantly changed cysteine oxidation (adjusted p-value < 0.05) between fetal and adult HSPCs and peptides with cysteines found to be oxidized only in fetus (3: 0 fetal: adult valid values; marked with red frames) that belong to 43S PIC. **b** Heatmap represents oxidation level (%) of unique cysteine peptides belonging to the 43S PIC that were significantly differentially oxidized after treatment with 100 μM and 500 μM H_2_O_2_ in hESC. **c** GO enrichment for proteins with cysteines significantly higher oxidized in hESC after treatment with 100 μM and 500 μM H_2_O_2_ for overrepresentation of biological processes. Redundant GO terms were filtered out. **d** Alteration to the eIF3B interactome upon H_2_O_2_ treatment in hESCs. The eIF3B interactome was co-immunoprecipitated (co-IP) from the whole cell lysates of hESC incubated with vehicle (Ctrl) or 100 μM H_2_O_2_ treated in two replicates with anti-eIF3B primary antibody or negative control. Proteins significantly (p-value < 0.05, Limma) differentially associating with the oxidized form of eIF3B are displayed in the heatmap. **e** Proteins identified in co-IP experiment were analyzed by STRING in Cytoscape. A functional protein association network was generated with the minimum required interaction score set to high confidence (0.7). Only significantly eIF3B-co-immunoprecipitated proteins (p-value < 0.05; enlarged circles) and their first neighbors are displayed. Red circles represent known subunits of 43S PIC. Node colors represent relative protein enrichment/depletion from the pulled down complex (log2 fold change (treated/ctrl)). The meaning of network edges was set to the term “co-expression” and network layout is edge-weighted spring embedded layout based on co-expression. **f** Log2 TE (polysome mRNA/total mRNA) for 100 μM H_2_O_2_-treated and untreated hESCs. Genes with p-value < 0.05 and log2 TE > 0.6 or <0.6 are depicted in red or blue. (For interpretation of the references to color in this figure legend, the reader is referred to the Web version of this article.)

**Fig. 5:**
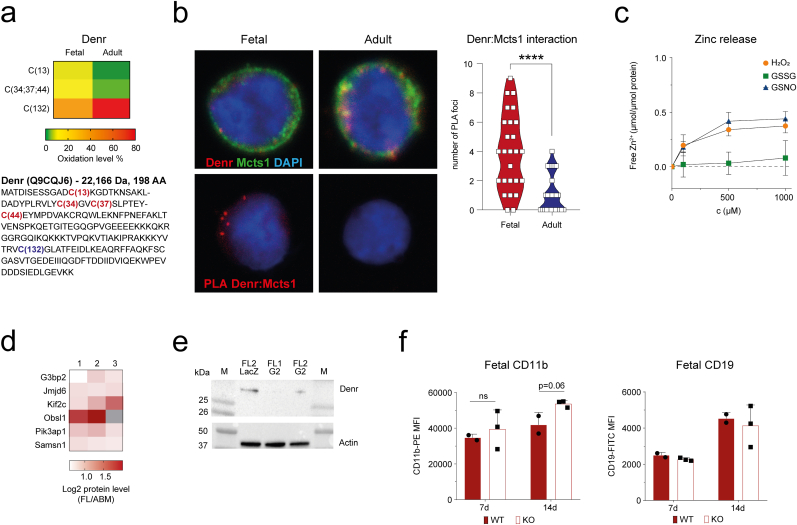
**Fetal hematopoiesis is regulated by a redox sensitive complex of translation re-initiation factors Denr and Mcts1.** **a** Heatmap depicting average oxidation level (%) of cysteine peptides that belong to translation re-initiation factor Denr in fetal and adult HSPCs. Statistics are shown in Table 1. Denr protein sequence with higher oxidized cysteines in fetus (red) and adult (blue) is shown. **b** Confocal images of in situ PLA foci (top) of Denr and Mcts1 translation re-initiation factors and proximity ligation assay (bottom) of Denr-Mcts1 complex in fetal and adult HSPCs using anti-Denr and anti-Mcts1 antibodies, and 4′,6-diamidino-2-phenylindole (DAPI) for DNA staining. Graph (right) depicts number of PLA foci per ten FL and ABM HSPCs. **c** Zinc release from zinc finger binding domain of Denr upon incubation with thiol reactive oxidative agents H_2_O_2_, nitrosylated glutathione (GSNO), and glutathione disulphide (GSSG). Each point represents the mean of three measurements. **d** Heatmap with log2 fold change expression level fetal/adult for targets of Denr-Mcts1 mediated translation re-initiation (Schleich et al., 2017) with significantly higher protein levels in fetal HSPCs compared to adult HSPCs (Jassinskaja et al., 2017). **e** Western blot depicting Denr and actin expression in fetal HSPCs after electroporation with Cas9 LacZ guide DNA (control) and Cas9 guide designed to disrupt expression of Denr. **f** Median fluorescence intensity (MFI) of myeloid (CD11b) (left) and lymphoid (CD19) (right) markers in wild-type (WT) and Denr knock-out (KO) fetal HSPCs cells after 7 and 14 days of culture. The bar graph presents mean ± SD in two or three biological replicates. ****p < 0.0001 (For interpretation of the references to color in this figure legend, the reader is referred to the Web version of this article.)

To investigate the sensitivity of 43S PIC subunits to thiol oxidation further, we administered hydrogen peroxide (H_2_O_2_) to an embryonic stem cell line (human embryonic stem cells (hESCs)) and performed a redox proteomic analysis using the same approach as for HSPCs (Fig. 1a). Similarly, we found that proteins with redox regulated cysteines in hESCs are significantly enriched for RNA processing and translation initiation (Fig. 4c, Supplementary Table 4). We observed that, with the exception of cysteines of Rps2, all cysteines of 43S PIC proteins that were significantly differentially oxidized between fetal and adult HSPCs underwent oxidation, most of them up to 30%, upon treatment of hESCs with 500 μM H_2_O_2_ (Fig. 4b). We found that cysteines of eIF3B (Cys373), -3D (Cys437 and Cys438) and -3I (Cys99) underwent oxidation in a concentration-dependent manner, acquiring similar or higher levels of oxidation as those observed in mouse HSPCs (Fig. 4b). Hence, the thiols of eIF3 are redox sensitive and are able to respond to concentrations of H_2_O_2_ that are physiologically recoverable for the cells [21]. The oxidation sensitivity of cysteines of eIF3D (Cys437 and 438) confirms that their higher oxidation in adult HSPCs (Fig. 4a) is under regulatory control (Fig. 4b and SF5b). We observed a similar ontogenic regulatory control within eIF3B protein sequence, with only one cysteine (Cys373) higher oxidized in fetal HSPCs, while the other three detected cysteines (Cys291, Cys409, Cys504) were higher oxidized in adult cells (although not significantly; Fig. 4a and SF5a, b). Motif analysis did not show that the redox regulated 43S PIC proteins are characterized by a common domain or motif that would make these proteins more susceptible to oxidation.

eIF3, the largest component of 43S PIC, plays a central role in the formation of the 43S PIC by acting as a scaffold orchestrating a multitude of interactions among several eIFs that assemble on the 40S subunit and participate in the different reactions involved in translation [38,39]. It has been demonstrated that some of the eIF3 subunits have regulatory functions such as controlling global protein synthesis rate and/or mediating the translation of subsets of mRNAs under specific conditions [40,41]. Thus, we investigated whether redox switching of cysteines on eIF3 proteins might affect their binding properties. We chose eIF3B specifically because 1) it interacts (directly or indirectly) with most of the eIF3 subunits and the other protein components of 43S PIC 2) the role of eIF3B is conserved in the structural organization of the eIF3 complex, 3) the redox sensitive Cys373 of eIF3B is localized in a WD repeat domain providing multiple protein-protein binding surfaces for reversible protein complex formation [42], as well as in the region known to be sufficient for interaction with eIF3E and 4) all identified cysteines of eIF3B in fetal and adult HSPCs, including Cys373, showed oxidation sensitivity in hESCs (Fig. 4a, SF5b). We performed co-immunoprecipitation of eIF3B in hESCs incubated with or without H_2_O_2_. We identified 257 proteins that met the criteria for a specific interaction with eIF3B, of which 24 are components of 43S PIC. We found 11 proteins to be differentially associated with the oxidized form of eIF3B compared to the untreated form (p-value <0.05; Supplementary Table 5, and Fig. 4d and e). These were all depleted from the complex following H_2_O_2_ treatment, suggesting altered eIF3B binding properties. Among the differentially associated proteins that were previously experimentally determined to directly interact with eIF3B and to be part of 43S PIC complex was RPS6, regulating selective translation of particular classes of mRNA [43]. In addition, eIF6, which binds to the 60S ribosomal subunit and is rate-limiting in translation initiation [44], and RPL11, a component of the 60S ribosomal subunit, were also found to be differentially associated with the oxidized form of eIF3B. We asked whether oxidative conditions affect global protein synthesis and/or selective translation. Through metabolic labelling experiment we found that H_2_O_2_ treatment of hESCs led to a slight increase in global protein synthesis (SF5c). Polysome profiling (SF5d) and sequencing of the actively translated mRNAs (polysome mRNAs) in H_2_O_2_-treated hESCs revealed only mild differences in translational efficiency (TE; polysome mRNA/total mRNA), which did not meet our FDR cutoff (FDR <0.05; Supplementary Table 2; SF5e). Nevertheless, the transcript with the lowest p-value was Ferritin light chain (FTL) (Supplementary Table 2), which is known to be translationally regulated and repression of its translation can be controlled by eIF3 [45]. Among the mRNAs with increased TE upon oxidative conditions (log2TE > 0.6; p < 0.05; Fig. 4g), we found GOLGA6L9, TAF13 and the hematopoietic cell fate modulator MEF2C [46].

Taken together, our data indicate that a pro-oxidative environment via cysteine oxidative modifications affects recruitment of binding partners to eIF3B; however, the overall effects on global protein synthesis and/or selective mRNA translation in hESCs are moderate.

### 2.5 Redox regulated translation re-initiation factors demonstrate higher levels of interaction in fetal compared to adult HSPCs

Protein production must be strictly controlled and eukaryotic organisms have developed many layers of translation regulation. One such layer, termed translation re-initiation, was evolved to modulate translation of a specific group of transcripts containing upstream open reading frames (uORF) with strong Kozak sequences (stuORFs) [47]. We identified changes to oxidation of peptides belonging to two re-initiation factors - Density regulated protein (Denr, Q9CQJ6) and Malignant T-cell-amplified sequence 1 (Mcts1, Q9DB27) (Table 1; Fig. 5a). Previous studies have shown that Denr and Mcts1 form a complex involving cysteine residues of Denr and that this complex binds the 40S ribosomal subunit to mediate translation re-initiation on transcripts containing stuORFs [47,48]. To test if differentially oxidized peptides of Denr affect its interaction with Mcts1 in fetal and adult HSPCs, we utilized a proximity labelling assay (PLA) to first compare the presence of Denr-Mcts1 complex in these cells. Intriguingly, we detected significantly higher level of Denr-Mcts1 complex in fetal compared to adult HSPCs (Fig. 5b). As it has been shown that the Denr-Mcts1 interaction is dependent on a zinc finger at the N-terminus, comprised of Cys34, Cys37, and Cys44 [48], we next tested the zinc finger stability upon cysteine oxidation. Upon treatment of Denr protein with different oxidative agents we confirmed disrupted zinc finger stability upon oxidation (Fig. 5c). However, as the oxidation of zinc finger-forming cysteines was higher in fetal relative to adult HSPCs (Table 1; Fig. 5a), we concluded that oxidation of cysteines participating in the zinc finger is an unlikely cause of the lower presence of Denr-Mcts1 complex in adult HSPCs. Instead, we hypothesized that the differential presence of Denr-Mcts1 complex relates to the observation that 84% of peptides containing Cys132 are oxidized in adult HSPCs, in contrast to only 36% oxidation in fetal cells (Fig. 5a). Since cysteines with high levels of oxidation have the capacity to form disulphides [22], we submitted the sequence to two independent disulphide prediction tools [49,50]. In both cases, Cys132 was predicted to be in a disulphide bonding state with high confidence. This indicates that adult Denr exists as a different proteoform and may thus acquire different structural properties that do not allow formation of the zinc finger and subsequent interaction with Mcts1.

**Table 1:** Oxidation of peptides belonging to Denr (Q9CQJ6) and Mcts1 (Q9DB27) in fetal and adult HSPCs.

Protein ID	protein name	Sequence	Cys position within protein	Average % OX FL	Average % OX ABM	q-value
Q9CQJ6	Denr	ATDISESSGADCK	C(13)	9.33	1.19	0.015
Q9CQJ6	Denr	VCGLATFEIDLK	C(132)	35.92	83.98	0.058
Q9CQJ6	Denr	VLYCGVCSLPTEYCEYMPDVAK	C(34; 37; 44)	14.29	3.86	0.065
Q9DB27	Mcts1	ENVSNCIQLK	C(14)	9.49	2.72	0.004
Q9DB27	Mcts1	FVLSGANIMCPGLTSPGAK	C(113)	11.95	9.00	0.512

We mapped described Denr targets [51] to the proteomic data from our previous study [2] and identified 6 proteins with significantly increased protein levels in fetal HSPCs whose translation is highly dependent on Denr-Mcts1 mediated translation re-initiation (Fig. 5d). Among these proteins Jmjd6, Pik3ap1 and Samsn1 play a role in the regulation of B lymphopoiesis, including B cell differentiation, activation and B cell receptor signaling [[52], [53], [54]]. To test the functional effect of the Denr-Mcts1 complex in fetal HSPCs, we used CRISPR-Cas9 to delete Denr (Fig. 5e) and determined the effects on *in vitro* differentiation. Although our data were not statistically significant, we observed a slight increase in expression of myeloid marker CD11b on Denr-deficient relative to wildtype (WT) cells (Fig. 5f; p-value = 0.06). The Denr-Mcts1 complex may therefore play a role in directing the cells towards B cell fate, possibly via translation of specific transcripts in fetal HSPCs.

Together, these findings suggest that Denr acquires different conformation via disulphide bond formation in adult HSPCs which have impact on its interaction with Mcts1, and highlight the possibility that fetal HSPCs employ Denr-Mcts1 complex for translation of proteins involved in B lymphoid differentiation.

### 2.6 Expression of leukemia-initiating fusion oncogene MLL-ENL is accompanied by significant cysteine oxidation in fetal HSPCs

Childhood leukemia, one of the main causes of cancer-related deaths in children, has been described to originate from fetal HSPCs [55,56]. Considering the oxidative burden that is associated with cancer [57], the increased mutational rate in fetal relative to postnatal hematopoiesis [58], and given the comparatively already high oxidation levels we found on fetal HSPC proteins, fetal proteins that show redox sensitivity to a leukemia initiating mutation may be compelling therapeutic targets. We therefore set out to identify cysteines with a change of oxidation state triggered during the early events of fetal leukemogenesis. We took advantage of a mouse model harboring doxycycline-inducible expression of the mixed-lineage leukemia-eleven-nineteen leukemia (MLL-ENL) fusion oncogene [59] to analyze the redox proteome in fetal HSPCs undergoing MLL-ENL-mediated leukemia initiation (Fig. 6a). We confirmed MLL-ENL expression following incubation with doxycycline (SF6a) and observed a significant impact on cellular phenotype upon MLL-ENL induction (Fig. 6b). Expression of the fusion oncogene was accompanied by significantly increased levels of oxidation of protein thiols, with median oxidation levels increasing to 20% upon MLL-ENL induction (Fig. 6c and d), emphasizing the oxidative sensitivity of the fetal cells. We identified 476 cysteine peptides (corresponding to 361 proteins) to contain redox-sensitive thiols with significantly higher oxidation in MLL-ENL-expressing compared to WT fetal HSPCs (SF6b). These proteins were enriched for amide metabolism and processes related to protein homeostasis (Fig. 6e), and included e.g. 6 components of the chaperonin-containing T-complex. 44 of the peptides overlapped with peptides having significantly differentially oxidized cysteines between fetal and adult HSPCs, of which 40 were higher oxidized in fetal cells (Fig. 6f). Intriguingly, we found proteins covering all the three classes of fetal signatures that we had revealed for redox-sensitive cysteines at steady-state: translation (Cys13-Denr, Cys138-eIF5 and Cys22-eIF5a), protein quality control system (Cys598/599-Hsp90aa1, Cys481-Uba1, Cys253-Psmd13 and Cys9-Psmd4) and energy metabolic proteins (Cys140-Ndufab1 and Cys172-Glud1; Fig. 6f and g). Hence, these ontogeny-specific oxidized thiols are targets of further oxidization upon MLL-ENL induction. In addition, cysteines of proteins regulating apoptosis and lymphocyte differentiation underwent oxidation upon MLL-ENL expression. One of these proteins was Casp3, for which thiol oxidation is known to suppress caspase-mediated apoptosis [60]. Connected to apoptosis and lymphocyte differentiation, we also found Lgals1 with increased oxidation upon MLL-ENL induction. Interestingly, Lgals1 function has been shown to be regulated by redox-dependent modulation of its structure [61]. We found Cys61 of Lgals1 to be higher oxidized in adult HSPCs, while it showed increased oxidation in fetal HSPCs upon MLL-ENL expression (Fig. 6f). Taken together, these data confirm that redox sensitive proteins undergoing oxidation in the more pro-oxidative environment of fetal HSPCs are prone to be higher oxidized upon genotoxic stress and leukemic transformation, and suggest a regulatory role for redox signaling acting in essential cellular processes, including protein homeostasis and metabolism, in leukemia development.

**Fig. 6:**
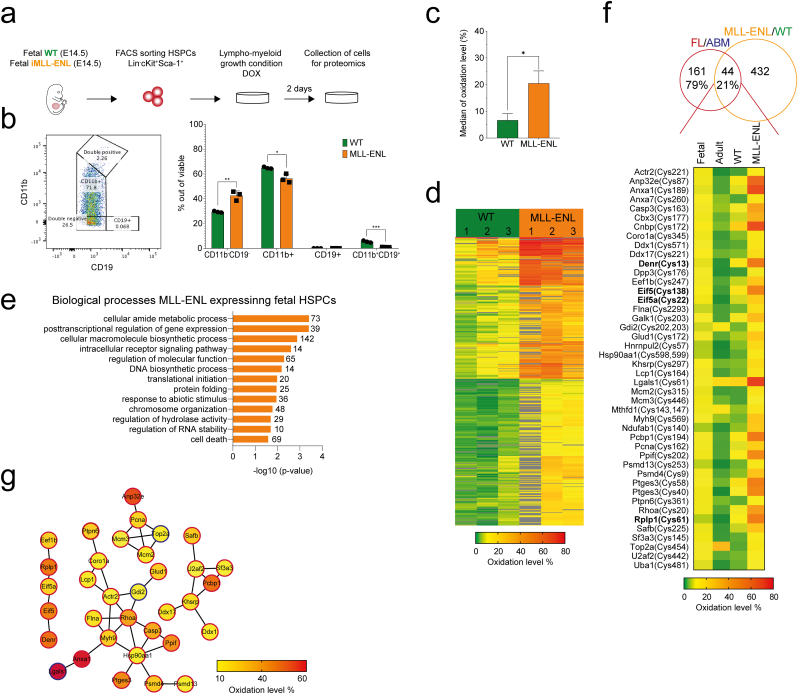
**Expression of the MLL-ENL fusion oncogene induces massive oxidation of protein thiols in fetal HSPCs**. **a***In vitro* workflow for MLL-ENL induction by doxycycline (DOX) in fetal HSPCs and collection of MLL-ENL expressing cells for proteomic analysis. **b** Representative FACS plot showing the gating strategy (left) and frequency of selected populations in MLL-ENL expressing cells (MLL-ENL) and wild-type controls (WT) (right). The bar graph shows mean ± SD in three biological replicates. **c** Median oxidation level for all quantified peptides in MLL-ENL and WT cells. The bar graph shows mean ± SD in three biological replicates. Statistical significance was evaluated by *t*-test with Holm-Sidak correction for multiple comparisons. **d** Heatmap represents % oxidation of identified and quantified peptides in MLL-ENL and WT cells in three biological replicates (1, 2, 3). **e** Gene ontology (GO) biological process enrichment for proteins with cysteines significantly higher oxidized upon expression of MLL-ENL oncogene in fetal HSPCs. Significantly enriched (p-value < 0.05) biological processes were selected and redundant GO terms were filtered out. **f** Overlap between 476 peptides significantly higher oxidized peptides in MLL-ENL cells compared to WT and 205 peptides significantly differentially oxidized in fetal compared to adult HSPCs. Average oxidation level for unique cysteine peptide from three biological replicates is shown in the heatmap. Positions of oxidized cysteines within protein sequence are indicated. Proteins involved in translation are highlighted in bold. **g** Protein network of 44 peptides higher oxidized upon MLL-ENL expression in fetal HSPCs and differentially oxidized between fetal and adult HSPCs analyzed by STRING in Cytoscape. The minimum required interaction score was set to 0.5. Nodes' border color represents peptides significantly higher oxidized in fetus (red) and adult (blue). Fill color of nodes corresponds to % of oxidation of peptides found in MLL-ENL expressing HSPCs. *p < 0.05, **p < 0.01, ***p < 0.001. (For interpretation of the references to color in this figure legend, the reader is referred to the Web version of this article.)

## 3 Discussion

In the presented study, we have studied ontogeny-specific PTMs of cysteines in primary mouse HSPCs, starting from ten-fold less protein amount relative to other recently published redox proteomic analyses [19,21,62]. Denaturing conditions and accessibility of label reagent are critical for the study of cysteine oxidations, as poor accessibility to the alkylating agent may impact artificial alkylations due to oxidation being faster than the alkylation step [63]. We used the MS-compatible detergent SDC as basis for the lysis buffer, because it showed superior cell lysis, protein extraction and solubilization after protein precipitation in comparisons to the detergent Rapigest SF (Waters) (data not shown). Because we detect physiological levels of oxidation, together with our close to complete labeling (SF2), we exclude the possibility of any bias introduced by poor accessibility of thiols to the alkylating agent. Our study provides a unique resource describing the redox state of 4,500 cysteine sites and their regulation during development of the blood system. We have taken great care to minimize artificially introduced oxidations during sample handling. Nevertheless, because of the inevitably rather long sample processing times to isolate primary HSPCs, and the sensitivity of cysteines to oxidation during sample preparation, our data reflects the oxidative state close to, but not fully, in vivo state.

We have identified numerous proteins where cysteines are more prone to oxidation or undergo reversible modification uniquely in fetal HSPCs. In contrast to protein expression-based proteome complexity, which we have previously shown to be considerably higher in adult HSPCs relative to fetal HSPCs [2], the cysteine redoxome is far more diverse in fetal cells relative to adult cells. This illustrates an especially important regulatory role for redox signaling in fetal hematopoiesis. The higher susceptibility of protein thiols to oxidation in fetal HSPCs is likely a result of differential redox homeostasis in fetal and adult HSPCs, characterized by higher oxidative state and lower levels of antioxidant enzymes in fetal compared to adult cells [2]. Our data indicate that this pro-oxidative environment in fetal HSPCs is an important contributor to redox regulation of large molecular machineries and fundamental processes such as protein homeostasis and metabolism that are known to be subject to considerable ontogenic remodeling in HSPCs [2,3,64]. At the same time, this distinct redox homeostasis makes fetal HSPCs more vulnerable to increased exposure to ROS, for example during genotoxic stress, where sensitive proteins can become targets of uncontrolled redox signaling or oxidative damage. In line with this, our data showed a substantial increase in the global protein oxidation upon MLL-ENL induction in fetal HSPCs. Potential sources of elevated ROS causing such increased cysteine oxidation remain to be investigated.

We found that mitochondria, in addition to the nucleus and cytoplasm, contain the highest percentage of proteins with low levels of oxidation, despite that, as for most cells, the main source of ROS in HSPCs can be expected to be the mitochondria [57]. This supports the proposed idea that mitochondria maintain their redox environment in more reducing conditions to prevent cell death [65]. Our results propose that such regulation is intertwined with thiol oxidations within the electron transport chain itself. It is tempting to speculate that the redox changes to thiols in fetal cells promote (rather than attenuate) mitochondrial ROS generation [66] and cause a higher activity of oxidative phosphorylation in fetal compared to adult HSPCs [10]. In line with this, the Complex I subunit Ndufab1, which we found to be sensitive to oxidation in fetal cells and with increased oxidation upon MLL-ENL-mediated leukemia initiation, confers greater capacity and efficiency of mitochondrial energy metabolism [67]. We also demonstrated that the spatial distribution of proteins based on cysteine oxidation levels was fairly equal between fetal and adult HSPCs. These findings support the redox hypothesis by demonstrating that the redox environment is spatially robustly controlled in the cell in order to provide an oxidative gradient specifically in the proximity of corresponding protein thiols to mediate cell signaling [68,69]. The reciprocal oxidation of individual cysteines in translation initiation factors 3b and 3d detected in fetal and adult HSCPs supports the redox hypothesis, and confirms that the increased number of proteins with oxidized cysteines in fetal HSPCs is not a mere consequence of increased ROS, and that our identified redox-sensitive cysteines are linked to resultant signals or other functional effects with developmental purpose.

The most prominent biological processes that appeared to be under control of redox signaling during hematopoietic development, such as energy metabolism or protein homeostasis, overlap with regulation of the protein levels in the same processes [2], however this is not apparent on the level of individual proteins. These findings point to the orchestration of both regulatory mechanisms and their crosstalk during ontogeny. The ubiquitin-proteasome system is the major cellular machinery responsible for the regulated degradation of cellular proteins to maintain cellular homeostasis [70]. Our findings of oxidation of specific Cys targets in the ubiquitin-proteasome system point to redox controlled alteration of proteasomal degradation in fetal HSPCs. Uba1 forms the first step in marking proteins with ubiquitin for proteasomal degradation and is a promising target of acute myeloid leukemia therapy [71]. While we had found all three catalytic subunits of the standard 20S proteasome to be strongly higher expressed in fetal HSPCs than adult HSPCs [2], all proteasomal redox-sensitive cysteines belonged to other parts of the constitutive 26S proteasome. The additional oxidations of regulatory cysteines in Uba1 suggest another level of regulation of proteasomal degradation contributing to the overall dynamic nature of FL HSPCs. Notably, many components of the ubiquitin-proteasome system are highly sensitive to oxidative stress, leading to uncoupling of the 26S proteasome and accumulation of ubiquitinated proteins [72]. On the other hand S-glutathionylation of alpha subunits of 20S proteasome is thought to cause higher degradation rates of oxidized proteins [73]. One should also consider that our approach does not detect the thiol modifications that are considered irreversible (sulfinic or sulfonic acid), for which the proteins are likely to be degraded by the proteasome. Further investigations are needed to resolve whether irreversible cysteine modifications form an additional layer of control of protein homeostasis in normal and malignant HSPCs. Intriguingly, the molecular chaperone Hsp90a that aids in the proper folding of specific client proteins, is known to target the N-terminal side of MLL, and we found Cys598/599-Hsp90aa1 with increased oxidation upon MLL-ENL leukemic initiation. Our results suggest that the oxidation of Cys598/599-Hsp90aa1 has a positive effect on its chaperone activity, making the fetal HSPCs more vulnerable to the MLL-ENL induction than the adult cells. This supports that inhibitors of Hsp90 may be a promising therapeutic approach that modulates the function of MLL fusion proteins in leukemic cells [74].

Our data highlights cysteine oxidation as contributor to altered mitochondrial dynamics as well as a shift in aerobic versus anaerobic glycolysis in HSPCs. Cysteine oxidation of metabolic proteins has previously been shown to be required for their proper function and represents one of the mechanisms of cellular adaptation to acute and chronic oxidative stress [21,75]. Several lines of evidence point towards a critical role for changes in cellular redox state, ROS levels and mitochondrial metabolism in regulating the balance between HSC self-renewal and differentiation [2,76] and targeting metabolic features has been proposed for selective eradication of leukemia stem cells [75,77]. Specifically, glutathionylation of Sdha, a key component of Complex II, has emerged as a promising therapeutic strategy to eradicate leukemic stem cells in adult leukemia patients [77]. Here, already two days after induction of expression of MLL-ENL in fetal HSPCs, we observed significantly increased cysteine oxidation of the Complex I component Ndufab1 together with 25 enzymes involved in energy metabolic pathways. We suggest that cysteine oxidation of these metabolic proteins may be drivers of the metabolic reprogramming to leukemic cells. However, further investigations are needed to delineate the functional consequences of oxidation of metabolic enzymes in fetal progenitors and the possibilities to use such strategy for therapy in childhood leukemia.

We found several components of the translational machinery to be differentially oxidized between fetal and adult HSPCs. While previous work showed that, in yeast and HEK 293 cells, redox switches of ribosomal proteins can mediate a decrease in global translation upon oxidative stress [19], our results point to a mild increase in translation in hESC treated with concentrations of H_2_O_2_ that are physiologically recoverable and thus rather represent steady-state oxidative signaling than oxidative stress [21]. We showed that eIF3B and eIF3D cysteines are similarly sensitive to increased ROS in our hESC model treated with H_2_O_2_, and demonstrated that modification of these cysteines modulates the binding properties of eIF3B. We speculate that modification of eIF3B cysteines might regulate translation via maintenance of large ribosomal subunit availability and selective translation of mRNAs containing 5′-terminal oligopyrimidine tract (TOP) motifs, respectively [43,78]. Although we lack direct proof of the impact of oxidation of eIF3B and eIF3D proteins on complex assembly in HSPCs, we expect that oxidation of initiation factors similarly affects protein conformation and binding properties in HSPCs as in hESCs, thus with potential to control selective translation and posttranscriptional regulation of blood cell development. Interestingly, recent work has revealed regulation of lineage commitment in HSPCs by ribosome levels and subsequent selection of transcripts for translation [79].

The lack of the Denr-Mcts1 complex in adult HSPCs led us to speculate that Denr-Mcts1-dependent translation re-initiation is important in HSPCs specifically during development. While 3 out of the 6 targets of Denr-Mcts1 mediated translation re-initiation that are higher expressed in fetal HSPCs are involved in B lymphopoiesis, deletion of Denr in fetal HSPCs only led to a very mild increase in expression of myeloid marker *in vitro*. It is therefore possible that Denr-Mcts1-mediated translation re-initiation is part of a larger regulatory network that exhibit effects on fetal HSPC cell fate. Denr- and Mcts1-dependent transcripts include several additional genes important for hematopoietic development, such as ATF4, G-coupled receptors [80,81] and AF1q (MLLT11) [47]. Moreover, AF1q is a known fusion partner of MLL [82]. It is believed that Denr-Mcts1 mediated re-initiation can occur only in specific tissues or during restricted periods throughout development as a consequence of modulated expression of Denr or Mcts1 proteins, or canonical initiation factors [83]. Our previous data showing no Denr or Mcts1 expression differences between fetal and adult HSPCs nor between more restricted progenitors [2,84], together with our findings here, suggest that oxidative PTMs play a role in such modulation as well. Further studies will be needed to shed more light on the role of oxidative signaling in regulation of translation initiation and translation re-initiation in normal and malignant hematopoiesis.

Taken together, applying the biological model of molecularly distinct fetal and adult HSPCs has allowed us to pinpoint important areas of redox regulation in developmental hematopoiesis and its direct link to leukemic transformation that may be exploited therapeutically.

## 4 Materials and methods

### 4.1 H9 hESC cell culture

H9 human embryonic stem cells (hESCs) with a normal 46, XX karyotype were acquired from the WiCell Research Institute (Madison, Wisconsin, USA). H9 cells were grown on Matrigel (VWR) coated plates and maintained in mTeSR™1 media (Stem Cell Technologies) following manufacturers’ instructions. H9 cells were incubated with vehicle, 100 or 500 μM H_2_O_2_ for 2 h at 37 °C.

### 4.2 Mice

Wild-type C57Bl/6 N mice purchased from Taconic Biosciences or bred in-house were used for all experiments. The inducible mouse model of MLL-ENL-driven leukemia (iMLL-ENL; *Col1a1*^TetO_MLL-ENL^) has been described previously [59]. All experiments involving animals were performed in accordance with ethical permits approved by the Swedish Board of Agriculture. Animals were housed in individually ventilated cages (IVC) and provided with sterile food and water *ad libitum*. Adult mice used in experiments were 10–20 weeks old. E14.5 embryos and neonatal mice were obtained by timed pregnancies overnight. The morning after mating was considered E0.5. For proteome analysis, equal numbers of male and female mice were used.

### 4.3 Flow cytometry and FACS

For redox proteomic experiments, cells were kept on ice and protected from light throughout the sample preparation. For all mouse flow cytometry and FACS experiments, single cell suspensions were prepared as follows. ABM was extracted from hind limbs, hip bones, forelimbs, shoulders, sternum, and spine collected in collection media (Hank’s Balanced Salt Solution [HBSS; HyClone]/10% bovine serum albumin [BSA]/2 mM ethylenediaminetetraacetic acid [EDTA]). Single-cell suspension of ABM was obtained by crushing bones using a mortar and pestle and passing cell suspensions through a 40 μm filter. Red blood cells were lysed by briefly incubating ABM cells with ammonium chloride solution (StemCell Technologies) on ice. Lineage-positive cells were removed from ABM cell suspension by depletion for Gr-1 (RB6-8C5; BioLegend), Ter119 (TER119; BioLegend), CD3 (145-2C11; BioLegend), B220 (RA3-652; BioLegend) and CD11b (M1/70; BioLegend) using biotin-conjugated antibodies with MACS anti-biotin beads using the autoMACS Pro Separator (Miltenyi Biotech). FLs were extracted from embryos collected at E14.5 gestation. Single-cell suspension of FLs was obtained by mechanic dissociation and passing through a 40 μm filter. Red cells were removed from FL cell suspensions by depletion for Ter119 using a biotin-conjugated antibody with MACS anti-biotin beads using the autoMACS Pro Separator. Fetal and adult cells were surface-stained with fluorophore-conjugated antibodies against Sca-1 (D7; BioLegend), c-Kit (2B8; BioLegend), Gr-1, Ter119, CD3, B220 and Flt3 (A2F10; BioLegend). Adult cells were additionally stained for CD11b. Briefly before analysis, cells were incubated with 7AAD (Merck) for viability staining. All flow cytometry and FACS experiments were performed on BD FACS AriaIIu (70 μm nozzle), BD FACS AriaIII (70 μm nozzle) or BD FACS LSR Fortessa instruments at the FACS Core Facility at Lund Stem Cell Center. For redox proteomic analysis, cells from each tissue (FL or ABM) were sorted on separate days (SF2a). The period of time from tissue extraction of mouse bone marrow or fetal liver to the end of the sort was approximately 8 h for both fetal and adult cells, without any consistent difference between the tissues. FACS-sorted cells were stored in 10% trichloroacetic acid (TCA) at −80 °C until sample preparation. For the flow cytometric analysis experiments fetal and adult cells were prepared in parallel and analyzed sequentially without systematic bias. Data analysis was performed in FlowJo (BD).

### 4.4 Collection of MLL-ENL expressing cells

60,000 FACS-sorted fetal LSK cells from iMLL-ENL and wild type mice were cultured in Opti-MEM + GlutaMax medium (Gibco) supplemented with 10% fetal calf serum (FCS; HyClone), 1% penicillin/streptomycin (Gibco) and 0.02% 50 mM 2-mercaptoethanol (Gibco) and 25 ng/mL stem cell factor (SCF), 25 ng/mL Flt3-ligand (Flt3l), 20 ng/mL interleukin (IL) −7, 10 ng/mL IL-6, 10 ng/mL granulocyte (G) -colony stimulating factor (CSF), 10 ng/mL granulocyte-macrophage (GM)-CSF, 10 ng/mL IL-3 and 0.1% doxycycline (to both iMLL-ENL and wild-type cells). After two days, cells were harvested, washed 3x with HBSS and 500,000 cells/replicate were collected for proteomic analysis and stored in 10% TCA at −80 °C until sample preparation.

### 4.5 qPCR validation of MLL-ENL expression

MLL-ENL gene expression was confirmed by qRT-PCR experiments as previously described [85]. Briefly, cells were lysed using RLT buffer and RNA purification was performed using an RNA purification kit (Nordic BioSite). SuperScript III First-Strand Synthesis System (Thermo Fisher) was used for cDNA synthesis, and qRT-PCRs were run with EvaGreen (Bio-Rad). Expression levels were normalized to B-actin. The following primers were used: forward: gcagatggagtccacaggat and reversed: cccagctctaacctcacctg.

### 4.6 Cell cycle

For cell cycle analysis cells were fixed and permeabilized using Transcription-Factor Buffer Set (BD) and intracellularly stained with fluorophore-conjugated antibodies against Ki67 (16A8, BD Biosciences) and pHH3 (HisH3S28-D6, Thermo Fisher). Briefly before analysis 4′,6-Diamidino-2-Phenylindole, Dihydrochloride (DAPI; Thermo Fisher) was added to the stained cells.

### 4.7 Thiol detection

For thiol staining, samples were divided in two. One sample was stained with N-Ethylmaleimide (NEM; Sigma Aldrich) for 20 min on ice protected from light. Both samples were then stained with Maleimide conjugated to AlexaFluor594 (Thermo Fisher) for 20 min on ice protected from light. A sample pre-stained with NEM was used as background.

### 4.8 Intracellular redox state detection and sorting

Cells were incubated with HBSS containing 4 μM CM-H_2_DCFDA (Thermo Fisher) at 37 °C for 30 min. Oxidative state in the lymphocyte population with eliminated doublets were gated as low, medium and high.

### 4.9 Sample preparation for redox proteomic analysis

Cells in 10% TCA corresponding to 400,000 cells/replicate were sonicated 5 × 15s and centrifuged. Firstly, naturally occurring free protein thiols were blocked by suspending pellet in lysis buffer (LB, 3% sodium deoxycholate [SDC]/200 mM triethylammonium bicarbonate [TEAB]/1 mM EDTA) with 4 mM of a first label of iodoTMT (“iodoTMT1”) and incubation for 2 h at 37 °C. Unreacted iodoTMT1 was removed by precipitation with acetone (1:4). Next, reversibly oxidized protein thiols were reduced with 5 mM tris(2-carboxyethel)phosphine (TCEP)/LB for 1 h at 50 °C followed by blocking of new free thiols with 4 mM of a second label of iodoTMT (“iodoTMT2”) for 2 h at 37 °C. Unreacted iodoTMT2 was removed by acetone precipitation. Labelled samples of FL and ABM were combined for each replicate and digested with LysC (Wako) (enzyme: protein = 1: 50 w/w) in 0.1% RapiGest/50 mM ammonium bicarbonate for 4 h followed by overnight digestion with trypsin (Promega; enzyme: protein = 1: 50 w/w). RapiGest was precipitated by addition of 0.5% trifluoracetic acid (TFA) and removed by centrifugation. An aliquot corresponding to 4 μg of sample was stored for full sample analysis (“Full fraction”). Remaining sample was enriched with immobilized anti-TMT antibody resin (Thermo Scientific) according to manufacturer's protocol, with slight modifications. Briefly, samples were diluted 5x with 1xTBS (add 450 μl), added to 200 μg washed resins with antiTMT (400 μl slurry) and incubated on end-to-end rotator for 2 h at 25 °C. Samples were then centrifuged and flow through fraction was stored. Resins with bound sample were washed 3 × 5 min with TBS, 3 × 5 min 1 x TBS, 3 × 5 min H_2_O. Samples were eluted with 4 column volumes of 50% ACN/0.4% TFA, frozen and dried using vacuum centrifugation overnight. The flow-through fraction containing peptides that did not react with anti-TMT was stored for analysis (“Flow-through fraction”). Enriched peptides were eluted with 50% acetonitrile (ACN)/0.4% TFA (“Enriched fraction”). All fractions were dried by vacuum centrifugation and cleaned up using in-house made C18 micro-spin columns. Samples were dissolved in 4% ACN/0.1% formic acid (FA) prior to analysis by LC/MS.

### 4.10 Sample preparation for proteomic analysis of eIF3 complexes

eIF3 complexes were co-immunoprecipitated from whole cell lysates of H9 hESCs using GammaBind G Sepharose (GE Healthcare) with anti-eIF3B (Santa Cruz Biotechnology) as primary antibody. The detailed co-immunoprecipitation (CoIP) protocol has been described previously [86]. Briefly, H9 hESCs grown in 6-well plates in mTESR medium on Matrigel were incubated with water (Ctrl) or 100 μM H_2_O_2_ for 2 h at 37 °C in two replicates. Cell were washed with PBS and lysed with buffer A (1% NP-40, 20 mM Tris-HCl [pH 7.5]), 50 mM KCl, 10 mM MgCl2, 1x Protease inhibitor cocktail (Roche) and 1 mM dithiothreitol (DTT). Cell lysates were divided in two and incubated with either GammaBind G sepharose pre-incubated with eIF3B antibody (IP) or non-specific IgG as a negative control (MOCK) overnight at 4 °C. Sepharose beads were washed with 200 mM HEPES and bound proteins were digested on beads as described previously with slight modifications [87]. Proteins were digested with 5 μg/ml trypsin in 200 mM HEPES/0.1% RapiGest for 7 h at 37 °C. Supernatant containing peptides was collected and proteins remaining on beads were further digested with 5 μg/ml trypsin in 200 mM HEPES/0.1% RapiGest/1 mM DTT for 1.5 h at 37 °C. Supernatant was collected and both supernatants were combined. Samples were completely evaporated, re-dissolved in 200 mM HEPES and labelled with TMT according to manufacturer’s protocol. Ctrl (IP), Ctrl (MOCK), 100 μM H_2_O_2_ (IP) and 100 μM H_2_O_2_ (MOCK) labelled peptides were combined in each replicate. Combined peptides were cleaned up using home-made C18 micro-spin columns. Samples were dissolved in 4% ACN/0.1% TFA prior to analysis by LC/MS.

### 4.11 LC/MS analysis

MS analyses for redox proteomics experiments were carried out on an Orbitrap Fusion Tribrid MS instrument (Thermo Scientific) equipped with a Proxeon Easy-nLC 1000 (Thermo Fisher) using a 120 min linear gradient separation followed by a synchronous precursor selection (SPS)-MS^3^ method. Injected peptides were trapped on an Acclaim PepMap C18 column (3 μm particle size, 75 μm inner diameter x 20 mm length, nanoViper fitting), followed by gradient elution of peptides on an Acclaim PepMap RSLC C18 100 Å column (2 μm particle size, 75 μm inner diameter x 250 mm length, nanoViper fitting) using 0.1% (v/v) FA in LC-MS grade water (solvent A) and 0.1% (v/v) FA in ACN (solvent B) as the mobile phases. Peptides were loaded with a constant flow of solvent A at 9 μl/min onto the trapping column and eluted via the analytical column at a constant flow of 300 nl/min. During the elution step, the percentage of solvent B was increased in a linear fashion from 5% to 10% in 2 min, followed by an increase to 25% in 85 min and finally to 60% in an additional 20 min. The peptides were introduced into the mass spectrometer via a Stainless-Steel Nano-bore emitter (150 μm OD x 30 μm ID; 40 mm length; Thermo Scientific) using a spray voltage of 2.0 kV. The capillary temperature was set at 275 °C.

Data acquisition was carried out using a data-dependent SPS-MS^3^ method. The full MS scan was performed in the Orbitrap in the range of 380–1580 m/z at a resolution of 120,000 at full-width-half-max (FWHM) using an automatic gain control (AGC) of 4.0e5 and a maximum ion accumulation time of 50 ms. The top ten most intense ions selected in the first MS scan were isolated for ion trap collision-induced dissociation MS2 (CID-MS2) at a precursor isolation window width of 0.7 m/z, an AGC of 1.5e4, a maximum ion accumulation time of 50 ms and a resolution of 30,000 FWHM. The normalized collision energy was set to 35%. The precursor selection range for MS^3^ was set to an *m*/*z* range of 400–1,200 in MS2. Orbitrap higher-energy collision-induced dissociation (HCD)-MS^3^ scans were acquired in parallel mode with SPS (ten precursors), a normalized collision energy of 55% and a resolution of 15,000 FWHM in a range of 100–500 m/z. The fragment ion isolation width was set to 2 m/z, the AGC was 1.0e5 and the maximum injection time 120 ms.

For co-immunoprecipitation experiments, MS analyses were carried out on an Orbitrap Exploris 480 MS instrument (Thermo Scientific) equipped with an UltiMate 3000 UHPLC system (Thermo Scientific). Peptides were trapped on an Acclaim PepMap 100C18 column (5 μm particle size, 0.3 mm inner diameter x 5 mm length, nanoViper fitting), followed by gradient elution of peptides on an EASY-Spray column PepMap RSLC C18 (2 μm particle size, 75 μm inner diameter x 200 mm length, nanoViper fitting) using 0.1% (v/v) FA in LC-MS grade water (solvent A) and 0.08% (v/v) FA in 80% ACN (solvent B) as the mobile phases. Peptides were loaded with a constant flow of solvent A at 10 μl/min onto the trapping column and eluted via the analytical column at a constant flow of 300 nl/min. Peptides were separated using a linear gradient from 4% to 30% of buffer B in 40 min, followed by an increase to 45% in 20 min. Separated peptides were electrosprayed into the instrument. The full MS scan was performed in the Orbitrap in the range of 350–1,500 m/z at a resolution of 120,000 at FWHM using AGC target of 1.0e7 and a 50 ms maximum ion injection time. The top twelve most intense ions selected in the first MS scan were isolated for Orbitrap HCD at a precursor isolation window width of 0.7 m/z, an AGC of 2.0e5 and a 50 ms maximum ion injection time, and a resolution of 30,000 FWHM. The HCD collision energy was set to 38%.

### 4.12 MS data analysis and bioinformatic analysis for redox proteomics

MS raw data from all redox proteomic experiments were processed with MaxQuant 1.5.6.5 [88] with the following settings: for data including MS^3^-based acquisition, ‘Reporter ion MS^3^’mode with quantification of cysteine-specific iodoTMT labels reporter ions was used; mouse fetal and adult HSPC MS data and H9 hESC data were searched using the built-in Andromeda search engine against the Swissprot mouse database (downloaded 2017.07.05; 25,170 protein entries) and human database (downloaded 2017.07.05; 20,160 protein entries) respectively, together with commonly observed contaminants and reversed sequences for all entries; methionine oxidation and N-terminal acetylation were set as dynamic modifications; trypsin/P was set as the enzyme in specific digestion mode, allowing two missed cleavage sites; no fixed modifications were considered; the ‘match between runs’ algorithm was applied; precursor and MS/MS fragment mass tolerance were set to 20 ppm and 0.5 Da, respectively; an FDR of 1% was required for identification at protein, site and PSM level; the minimum number of 1 unique peptide for a protein group to be considered as identified and reported in the final table; the minimum ratio count for protein quantification was set to two and razor and unique peptides were considered for protein quantification. Full, enriched and flow-through fractions of each replicate were jointly processed. Modification peptide table from MQ output was processed as follows: Data was filtered for contaminants, peptides identified only by site and non-cysteine peptides. In addition, hits with missing quantitative information for both free (SH) and oxidized (Sox) thiols were removed. Reporter ion intensities were then log2 transformed. Data were normalized by adjusting the median of each channel to the median of the medians of each condition (fetal and adult) representing either free (SH) or oxidized (Sox) thiols, respectively. Normalized log2 data were non-transformed, and the sum of SH and Sox quantitative values were calculated for each ID. Missing values were replaced by 0 for SH and “NA” for Sox, and oxidation level was calculated as [Sox/(SH+Sox)]*100. After confirming normal distribution of the data (evaluated by the symmetrical distribution of the Gaussian curve in diagnostic plots of oxidation levels), the p-value for each peptide with at least 2 oxidation level valid values in each group (fetal, adult) was determined using a two-sided *t*-test. Then the permutation-based FDR correction implemented in Perseus (version 1.6.6.0) for all peptides was applied [89]. Changes in oxidation level with an adjusted p-value < 0.05 were considered differential. Peptides with three valid values representing Sox in fetal HSPCs and no valid value representing Sox in adult HSPCs were considered as oxidized only in fetal HSPCs (“FL only”). No peptides meeting these criteria were found in adult HSPCs. The PANTHER classification system was used to retrieve classification of proteins [90]. For proteins with differentially oxidized cysteines, the leading protein of the protein group was used in GO enrichment analysis using the functional annotation tool DAVID [91]. Redundant GO terms were filtered out using REVIGO [92]. Cytoscape (version 3.8.1) was used to retrieve information about subcellular localization. For proteins with multiple localization, the highest score determined the final localization. If two localizations scored equally, both were considered [93]. ClueGO Cytoscape application was used for generation of functionally grouped annotation network of protein levels and protein oxidations [29]. Perseus and Prism (GraphPad) were used for data visualization. Previously described reversible modifications were annotated from RedoxDB [94]. Known disulphide bond annotations were retrieved from UniProt (December 14, 2020) and the positions of disulphide bond-forming cysteine residues within the protein sequence were matched to the respective positions of cysteine residues within the tryptic peptides. In the case of peptides containing more than one cysteine residue, a disulphide bond annotation was assigned if at least one cysteine residue within the peptide was annotated as disulphide bond-forming in the database. For disulphide bond prediction, proteins were analyzed by disulphide prediction tools DiANNA and DISULFIND [49,50]. The mass spectrometry proteomics data and MaxQuant output text files have been deposited to the ProteomeXchange Consortium via the PRIDE [95] partner repository with the dataset identifiers PXD030043 (FL/ABM experiment) and PXD033115 (H9 hESC experiment).

### 4.13 MS data analysis and bioinformatic analysis of IP proteomic experiments

H9 hESC data from co-immunoprecipitation experiments were searched against the Swissprot human database (downloaded 2017.07.05; 20,160 protein entries) together with commonly observed contaminants and reversed sequences for all entries. Methionine oxidation and N-terminal acetylation were set as dynamic modifications. Trypsin/P was set as the enzyme in specific digestion mode, allowing two missed cleavage sites. An FDR of 1% was required for identification at protein, site and PSM level. Protein groups were filtered for contaminants, reverse hits, proteins identified only by site and at least 2 valid quantitative values. No fixed modifications were considered. Precursor and MS/MS fragment mass tolerance were set to 20 ppm and 0.5 Da, respectively. The minimum ratio count for protein quantification was set to two, and razor and unique peptides were considered for protein quantification. Unspecific interactors (log2 (IP/Mock) < 1) and proteins without quantitative value were filtered out, resulting in a dataset of 257 proteins. Statistical analysis was performed on ratios of normalized intensities using the Limma package in R/Bioconductor [96]. Proteins with a p-value < 0.05 were considered to be differentially pulled down. A functional protein association network was generated in Cytoscape (version 3.8.1) using the STRING application (version 1.6.0). The minimum required interaction score was set to “high confidence” (0.7) [93]. The meaning of network edges was set to the term “co-expression” and the network layout to edge-weighted spring embedded layout based on co-expression. The mass spectrometry proteomics data and MaxQuant output text files have been deposited to the ProteomeXchange Consortium via the PRIDE [95] partner repository with the dataset identifier PXD030046.

### 4.14 Western blots

Cells were washed with ice-cold PBS and lysed in ice-cold RIPA lysis buffer (150 mM NaCl, 1% NP-40, 0.5% sodium-deoxycholate, 0.1% sodium dodecyl sulphate, 10 mM TrisHCl [pH 8]) supplemented with protease inhibitor cocktails (Sigma). Lysates were cleared by centrifugation at 15,000 rpm for 15 min at 4 °C and supernatants were removed and assayed for protein concentration by BCA (Thermo Fisher). Equal amounts of proteins (at least 20 μg) were subjected to SDS-PAGE using 4–20% MiniProtean TGX Precast Protein Gels (Bio-Rad) and transferred to PVDF membranes (Bio-Rad). Mouse b-Actin (Sigma), rabbit b-Tubulin (Sigma), mouse DENR (Santa Cruz Biotechnology) and mouse eIF3B (Santa Cruz Biotechnology), and goat anti-rabbit IgG HRP (Abcam) and rabbit anti-mouse IgG HRP (Abcam) were used as primary and secondary antibodies, respectively.

### 4.15 Global measurements of protein synthesis

Cells were washed twice in PBS and starved for 40 min in DMEM methionine- and cysteine-free media, 10% dialyzed FBS and 1% penicillin/streptomycin. Cells were subsequently treated for 1 h with 30 mCi/mL protein labelling mix (EasyTag Protein Labelling Mix; PerkinElmer). Cell pellets were washed twice in PBS and harvested for protein analysis by Western blot. For all analyses, cell lysates were prepared using standard procedures and equal amounts of total protein were separated on a 10% SDS polyacrylamide gel and transferred to PVDF membranes. Membranes were exposed to autoradiography film (GE Healthcare) at −80 °C for 12–48 h. ^35^S methionine/cysteine incorporation was quantified using image software to analyze signal intensity and normalized to b-actin levels. All media and supplements were purchased from Thermo Fisher if not otherwise stated.

### 4.16 Proximity ligation assay and quantification of proximity ligation foci

PLA was performed in accordance with manufacturer’s protocol (Duolink In Situ; Sigma Aldrich). Briefly, FL and ABM HSPCs were fixed by incubation with chilled methanol for 10 min at −20 °C. Post-fixation, cells were incubated with ice-cold acetone at −20 °C for 2 min. After blocking in a humified chamber at 37 °C with Blocking Reagent (Duolink In Situ kit) for 30 min, primary antibodies against DENR (Santa Cruz Biotechnology) and MCTS1 (Novus Biologicals) were added at a concentration of 1:200 and incubated overnight at 4 °C. Anti-mouse PLUS and anti-rabbit MINUS probes (1:500; Duolink; Sigma Aldrich) were used for detection of primary antibody binding. Ligation and amplification were performed in accordance with the manufacturer’s instructions. Cells were washed and DNA was stained with DAPI to visualize nuclei. Images of the nuclei and proximity ligated foci were acquired using a Zeiss 780 Confocal Laser Scanning Microscope. Analysis of number of foci per cell was performed in ImageJ software in a non-blinded manner.

### 4.17 Immunofluorescence

FL and ABM cells were fixed in 4% formaldehyde and permeabilized in 0.1% Triton X100 in PBS (0.1% Tx100). Non-specific antibody binding was blocked for 1 h by incubation with 3% BSA in 0.1% Tx100. Cells were stained with primary antibodies against DENR and MCTS1 in blocking buffer overnight at 4 °C. After three 10 min washes with PBS, samples were incubated with secondary antibody conjugated to AlexaFluor488 or AlexaFluor594 fluorophores (Invitrogen) at room temperature for 2 h. After subsequent PBS washes, slides were mounted in ProLong Gold antifade reagent (Thermo Fisher Scientific) and scanned with a Zeiss 780 Confocal Laser Scanning Microscope.

### 4.18 Polysome profiling

Polysome fractionation was performed as previously described [97,98] with some modifications. Briefly, three sub-confluent wells of a 6-well plate of hESCs were used for total and polysome RNA isolation. Cells were washed with PBS and incubated for 10 min at 37 °C in media supplemented with 10 μg/mL cycloheximide (CHX; Sigma-Aldrich). Subsequently, cells were placed on ice, washed with ice-cold PBS containing 10 μg/ml CHX and lysed in 600 μL passive lysis buffer consisting of 10 mM Tris-HCl (pH 8.0), 150 mM NaCl, 1.5 mM MgCl2, 0.25% NP-40, 0.1% Tx100, 32 U/ml SUPERase-In RNase Inhibitor (Thermo Fisher), 150 μg/ml CHX and 20 mM DTT. Lysates were incubated on ice for 40 min with vortexing every 10 min and cleared by centrifugation at 18,000×*g* for 10 min at 4 °C. 100 μL of lysate was separated and used for total RNA isolation with 600 μL TRIzol and Direct-Zol RNA Miniprep (Zymo Research). Lysates were layered onto a linear sucrose gradient: 10%–60% sucrose (w/v), 25 mM Tris-HCl pH 7.4, 25 mM NaCl, 5 mM MgCl_2_, 0.1 mg/mL Heparin and 2 mM DTT in nuclease-free water and centrifuged in a SW41Ti rotor (Beckman) for 2.5 h at 40,000 rpm at 4 °C without brake. Fractions were collected and polysome profiles generated using a BioComp Gradient Station (BioComp). 800 μL of TRIzol was added to each 650 μL fraction and samples were immediately snap frozen on dry ice. Fractions 12, 13 and 14 containing heavy polysomes (more than 3 ribosomes) were pooled and RNA isolation was carried out using Direct-Zol RNA Microprep (Zymo Research). Sequencing libraries were generated using 500 ng of total or polysome RNA with the TruSeq Stranded Total RNA Library Prep Kit (Illumina) according to manufacturers’ instructions. Libraries were sequenced on an Illumina NextSeq 500 machine with single-end 75 cycles run using NextSeq 500/550 High Output v2 kit (Illumina).

### 4.19 Polysome-seq analysis

Reads were aligned to rRNA reference using Bowtie (v1.1.2) [99]. The rRNA alignments were discarded to reduce rRNA contamination. The unaligned reads were aligned to the hg38 human genome using STAR v2.7.3a [100] with the parameters "--outFilterMultimapNmax 1" for obtaining the uniquely mapped reads, and "--twopassMode Basic" for using the two-pass mapping mode. HTSeq (v0.9.1) [101] was used to count the number of reads per gene. Differential expression and differential ribosome association analyses were performed using the Bioconductor package DESeq2 [102] with an interaction term model [103]. Genes with an adjusted p-value < 0.1 were considered for classification into different regulation classes. Normalized counts of polysome mRNA from control and 100 μM H_2_O_2_ treated sample in three replicates were submitted to gene set enrichment analysis (GSEA) using GSEA pre-ranked software [104]. The following parameters were implemented to calculate normalized enrichment scores (NES) and false-discovery rates (FDR): *t*-test for ranking the genes and 1,000 permutations.

### 4.20 Detection of Denr zinc release

Recombinant Denr protein production was performed in strain *Escherichia coli* TUNER(DE3)/DENR_pET-26b(+) grown in BD Difco LB broth Miller, 30 °C, 250 rpm with 1 mM IPTG for 3 h. After cell lysis and ultracentrifugation, DENR was purified on a 5 ml HisTrap HP column. Peak fractions were pooled and digested with tobacco etch virus (TEV) protease. After TEV digestion, the protein solution was run a second time on the HisTrap column, and the flow through and wash fractions were pooled, concentrated and run on a HiLoad 26/600 Superdex 75 pg gel filtration column. Then, the protein was concentrated and snap-frozen in liquid nitrogen. A fraction from the gel filtration was analyzed by analytical size exclusion chromatography. Recombinant protein Denr was incubated for 2 h with thiol reactive oxidative agents (H_2_O_2_, nitrosylated glutathione [GSNO] and glutathione disulphide [GSSG]) and zinc release was detected by spectrophotometry using 4-(2-pyridylazo)resorcinol (PAR).

### 4.21 CRISPR/Cas9 knock-down of Denr in fetal HSPCs

DENR knock-down HSPCs were generated using the CRISPR/Cas9 genome editing system [105]. Sorted fetal HSPCs were cultured in serum-free expansion media (SFEM; Stem Cell Technologies) supplemented with 50 ng/ml Flt3l, 50 ng/ml IL-6, 10 ng/ml IL-3 and 50 ng/ml SCF for 2 days. After 2 days, 50,000 cells/condition were mixed with ribonucleoprotein complexes (Alt-R CRISPR-Cas9 sgRNA and Alt-R S.p. Cas9 nuclease V3) and electroporated using BTX nucleofector/electroporator for 5 ms at 125 V. Electroporated cells were cultured in 48-well plates seeded with 10,000 OP9 cells per well in Opti-MEM + GlutaMax medium (Gibco) supplemented with 10% FCS, 1% penicillin/streptomycin and 0.02% 50 mM 2-mercaptoethanol, and 25 ng/mL SCF, 25 ng/mL Flt3l, 20 ng/mL IL-7, 10 ng/mL IL-6, 10 ng/mL G-CSF, 10 ng/mL GM-CSF and 10 ng/mL IL-3 for 7 and 14 days. All cytokines were purchased from Stem Cell Technologies. Prior to flow cytometric analysis, cultured cells were incubated with FC-block and stained with fluorophore-conjugated antibodies against NK1.1 (PK136; BioLegend), B220, Gr-1 and CD19 (6D5 or 1D3/CD19; BioLegend) for investigating the lineage-potential of fetal HSPCs. Progenitor output was analyzed by FACS and expression of Denr was assessed by Western blot using anti-DENR (Santa-Cruz, cat # sc-136254).

## Data availability

The mass spectrometry proteomics data and MaxQuant output text files have been deposited to the ProteomeXchange Consortium via the PRIDE partner repository [95] and can be accessed as described above.

## Author contributions

J.H. and K.P. designed the study with input from M.J. J.H. supervised the project. K.P. designed and performed all the experiments with assistance from M.J. M.C. contributed to PLA experiment. R.M. performed polysome profiling and sequencing experiments. P.C.T.N. performed bioinformatic analysis of sequencing data. N.G. contributed to metabolic labelling experiments. M.V. and N.G. contributed to work with H9 cells. C.B. designed and supervised experiments performed by R.M, P.C.T.N and N.G. E.J., M.V. and M.J. assisted with animal work. K.P. prepared the manuscript together with J.H. and M.J and critical input from all authors. J.H. and K.P. acquired funding.

## Conflict of interest disclosure

The authors declare no competing financial interests.

## Figures and Tables

**figs1:**
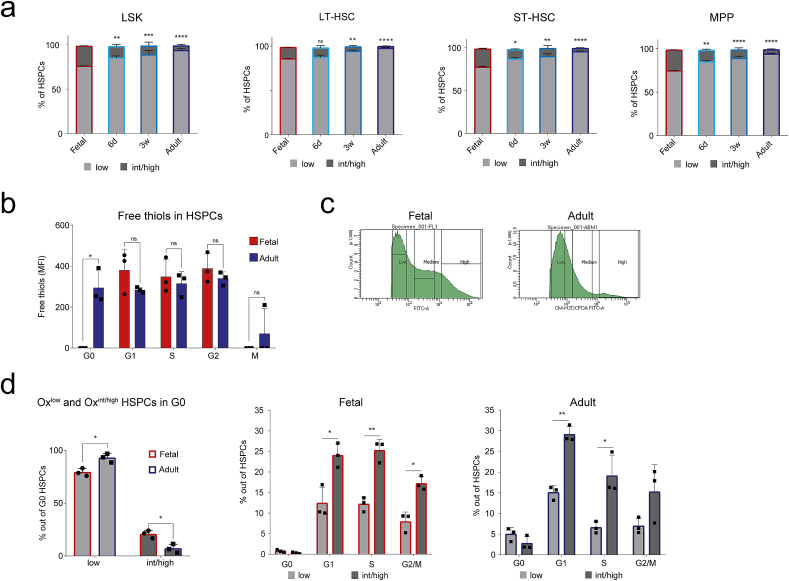


**figs2:**
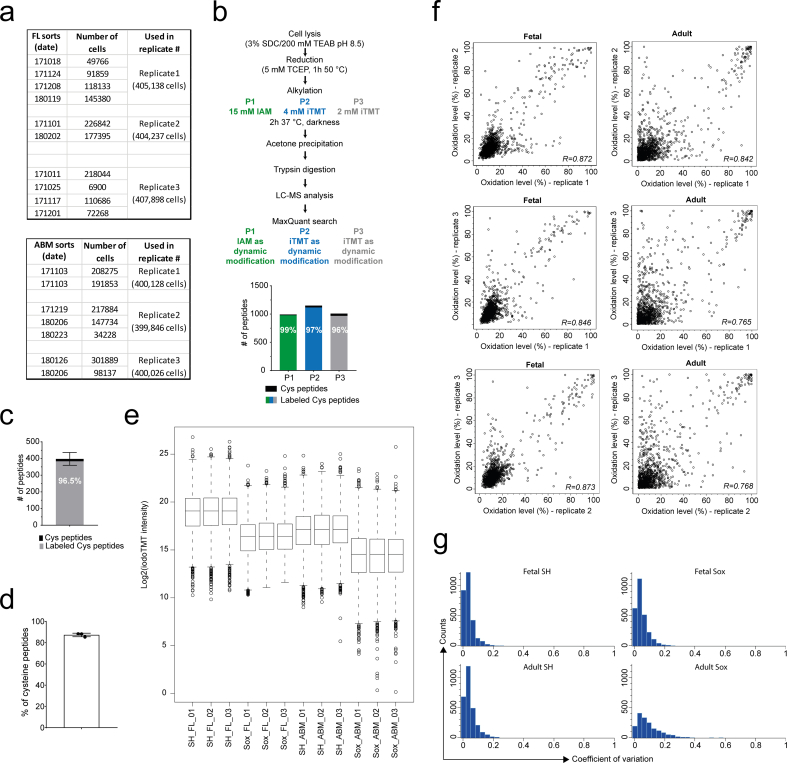


**figs3:**
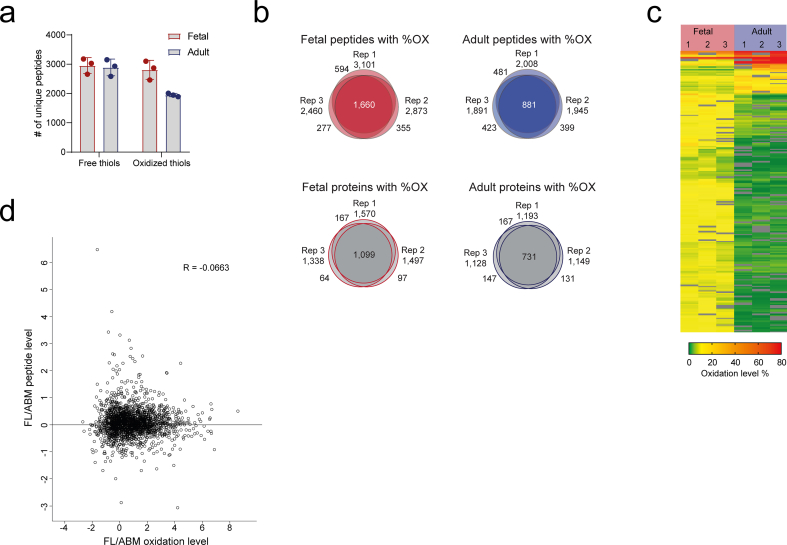


**figs4:**
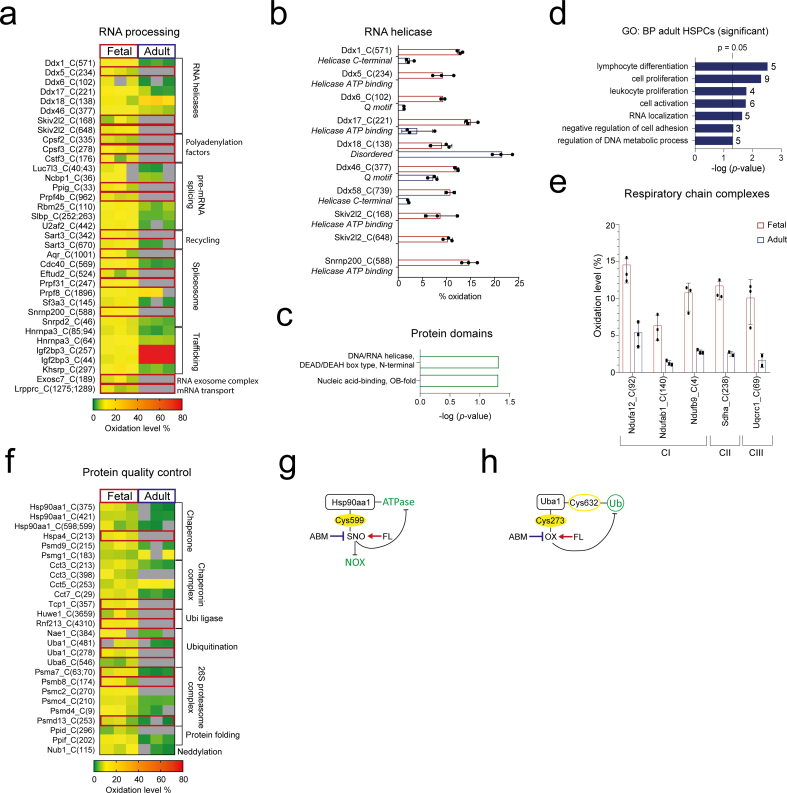


**figs5:**
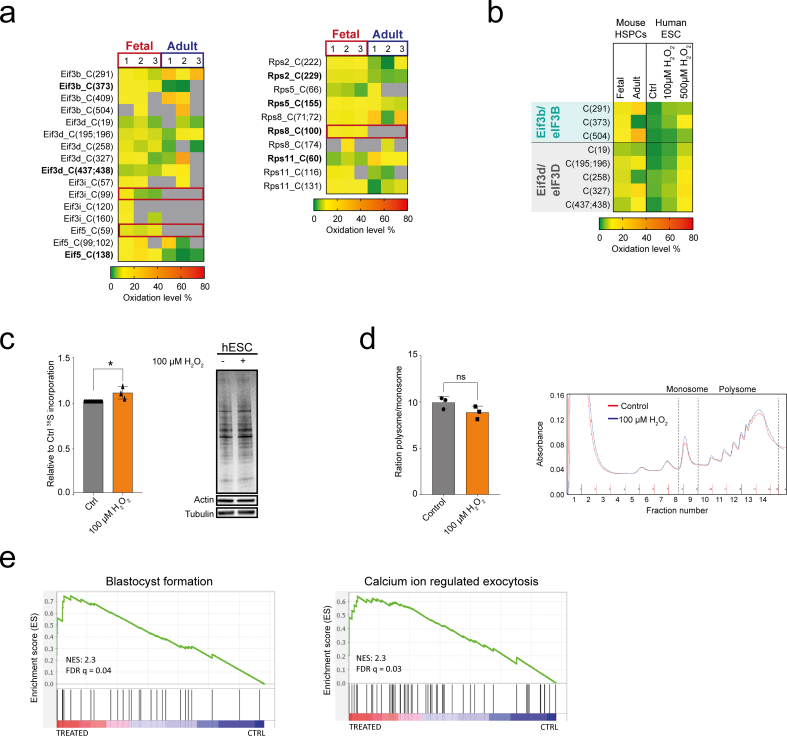


**figs6:**
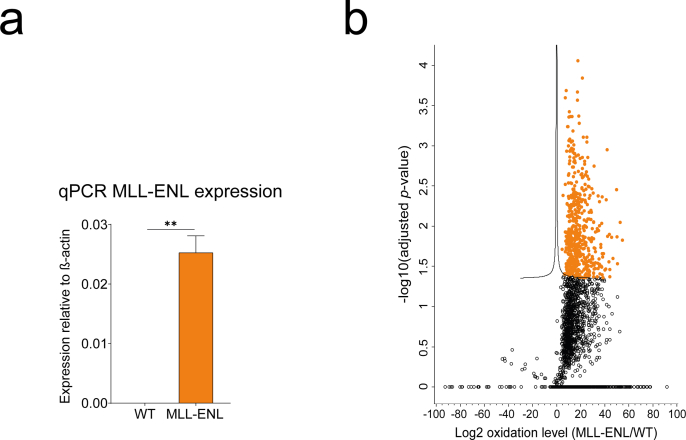


**figs7:**
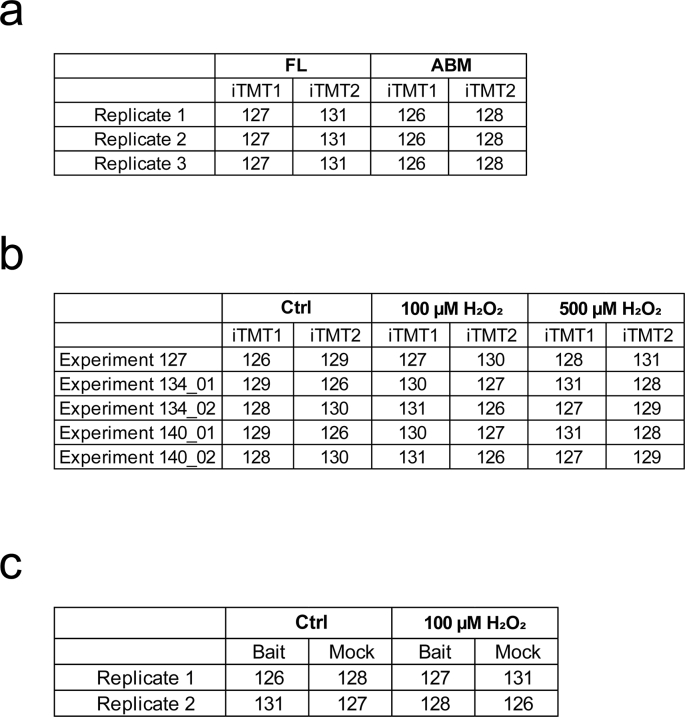


**figs8:**
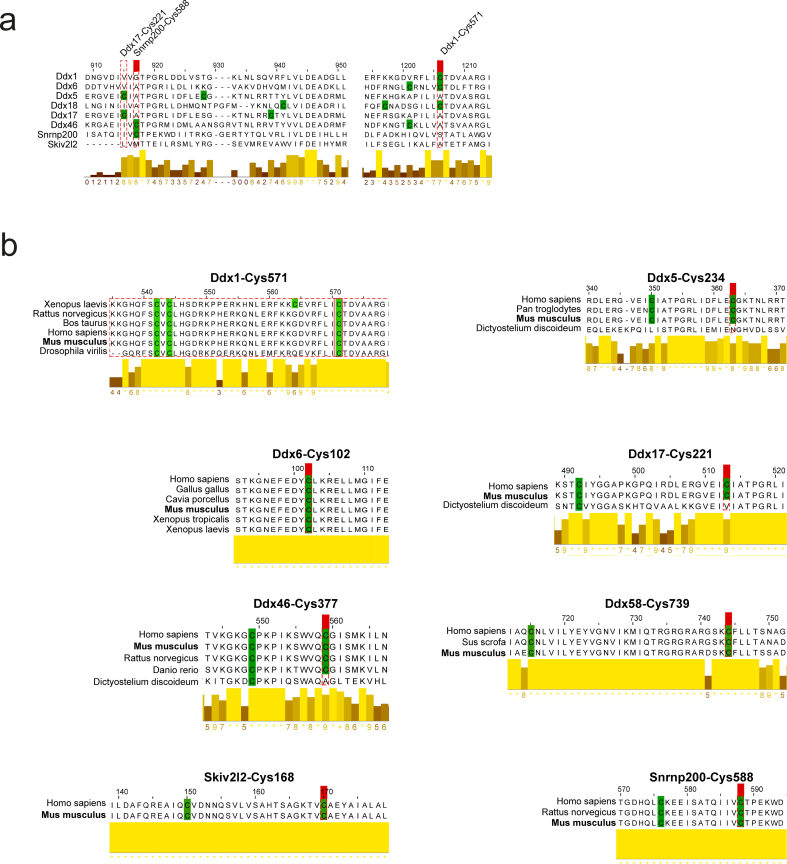


## Supplemental material

The article includes eight Supplemental Figures and six Supplemental Tables.
