# Supplementary material for: Quantitative analysis of redox proteome reveals oxidation-sensitive protein thiols acting in fundamental processes of developmental hematopoiesis
Source: Redox Biol. 2022 May 23;53:102343. doi: 10.1016/j.redox.2022.102343 (PMC9157258; doi:10.1016/j.redox.2022.102343)
Supplement: Multimedia component 1 [file mmc1.docx]

**Supplementary tables and legends**

**Supplementary Table 1.** Redox proteome data for peptides identified in fetal liver (FL) and adult bone marrow (ABM) mouse hematopoietic stem and progenitor cells (HSPCs).

**Supplementary Table 2.** RNA sequencing of polysomal and total fraction of human embryonic stem cells H9 treated with 100 µM H_2_O_2_ or vehicle (CTRL).

**Supplementary Table 3.** Peptides with redox sensitive cysteines identified in fetal liver (FL) and adult bone marrow (ABM) mouse hematopoietic stem and progenitor cells (HSPCs).

**Supplementary Table 4.** Redox proteome data for peptides identified in human embryonic stem cells H9 treated with 100 µM and 500 µM H_2_O_2_ or vehicle (CTRL).

**Supplementary Table 5.** Proteomic data of co-IP of eIF3B in human embryonic stem cells (hESC) treated with 100 µM (Ox) or vehicle (CTRL).

**Supplementary Table 6.** Redox proteome data for peptides identified in mouse fetal hematopoietic stem and progenitor cells (HSPCs) expressing fusion oncogene MLL-ENL (L) and wild-type HSPCs (WT).

**Supplementary figures and legends**

**Supplementary Figure 1.** **a** Distribution of cells with low (Ox^low^) and intermediate/high (Ox^int/high^) oxidative state within the Lin- Sca+ cKit+ (LSK), long-term HSCs (LT-HSC), short-term HSCs (ST-HSC) and multipotent progenitors (MPP) populations at E14.5 (Fetal), postnatal day 6 (6d), 21 (3w) and 10-20 weeks of age. Cellular oxidative state was evaluated by flow cytometry using CM-H_2_DCFDA. Data depicted is mean ± SD of 2-4 independent experiments. **b** Levels of free thiols in fetal liver (Fetal) and adult bone marrow (Adult) hematopoietic stem and progenitor cells (HSPCs) at the different stages of the cell cycle. Data represents mean ± SEM of three independent experiments. **c** Gating strategy of Ox^low^ and Ox^int/high^ subpopulations for fetal liver (Fetal) and adult bone marrow (Adult) hematopoietic cells. **d** Proportion of Ox^low^ and Ox^int/high^ populations within fetal liver (Fetal) and adult bone marrow (Adult) HSPCs in different stages of the cell cycle. Ox^low^ and Ox^int/^high populations of fetal liver and adult bone marrow hematopoietic cells were first sorted by flow cytometry using CM-H_2_DCFDA (c). Cell cycle stages of LSK HSPCs of sorted Ox populations were evaluated by flow cytometry using Ki67, DAPI and pHH3. Data depicted is mean ± SD of three independent experiments. **^a^**Statistical significance was assessed by 2-way ANOVA with Dunnett’s multiple comparisons test. **^b,d^**Statistical significance was evaluated by t-test with Holm-Sidak correction for multiple comparisons. *p<0.05, **p<0.01, ***p<0.001, ****p<0.0001 and ns=non-significant.

**Supplementary Figure 2.** **Approach performance.** **a** Tables demonstrate date and number of individual flow cytometry sorts of fetal liver (FL) and adult bone marrow (ABM) HSPCs and their pooling into replicates 1, 2 and 3. **b** Number of cysteine peptides identified in separate experiment to test potential of iodo tandem mass tag (iTMT) to block free thiols. Three separate workflows (P1, P2 and P3) were applied using 50 µg protein. First cells were lysed using 3% sodium deoxycholate (SDC) and 200 mM triethylammonium bicarbonate (TEAB), reversibly modified thiols were reduced with 5 mM tris(2-carboxyethyl)phosphine (TCEP) and free thiols were blocked either with 15 mM iodoacetamide (IAM) (P1), 4 mM (P2) or 2 mM (P3) iTMT. Samples were trypsinized and analyzed by nano-liquid chromatography (LC) with tandem mass spectrometry (MS). Data were analyzed by MaxQuant appropriate dynamic modification. Bar plot shows number of unique cysteine peptides (black) and number of peptides carrying IAM (green) or iTMT modification (blue, grey) respectively. % of labeled peptides is indicated. **c** Flow through (FT) of redox proteomic analysis of fetal and adult hematopoietic stem and progenitor cells (HSPCs) were searched with MaxQuant setting iTMT as a dynamic modification. Bar plot displays average of Cys peptides (black) and iTMT labeled Cys peptides identified in three biological replicates with standard deviation. % of labeled peptides is indicated. **d** Bar chart depicting percentage of cysteine peptides out of all peptides identified in the enriched fraction from redox proteomic analysis in three biological replicates of fetal and adult HSPCs. **e** Normalized reporter ion log2 intensities representing free (SH) and oxidized thiols (Sox) in three biological replicates (01, 02, 03) of fetal and adult HSPCs. **f** Oxidation level (%) of unique cysteine-containing peptides quantified in each biological replicate in fetal (left) and adult (right) HSPCs. Pearson correlation coefficients are shown in the lower right corner. **g** Histogram of coefficients of variation of normalized reporter ion intensities representing either free (SH) or oxidized (Sox) thiols in three biological replicates in fetal and adult HSPCs.

**Supplementary Figure 3.** **a** Bar graph depicts number of unique peptides with valid quantitative value of iodoTMT reporter ion representing free thiols (Free thiols) or reversibly oxidatively modified thiols (Oxidized thiols) identified by MS in fetal and adult HSPCs in three replicates. **b** The number and overlap of unique cysteine peptides and unique cysteine proteins with valid value of oxidation level in three biological replicates in fetal and adult HSPCs. **c** Heatmap depicting oxidation level (%) of all peptides in fetal and adult hematopoietic stem and progenitor cells (HSPCs) in three biological replicates (1, 2, 3) identified by redox proteomic analysis. **d** Scatter plot depicting correlation of peptide oxidation level (%) fold change with protein expression fold change detected by Jassinskaja et al. [1] in fetal and adult HSPCs.

**Supplementary Figure 4. Redox modulation of HSPC proteins forms an additional layer of regulation in fetal cells. a** Heatmap represents oxidation level (%) of peptides with significantly changed cysteine oxidation (adjusted p-value < 0.05) and peptides with cysteines found to be oxidized only in fetus (3: 0 fetal: adult valid values; marked with red frames) that are involved in mRNA processing in three biological replicates in fetal and adult HSPCs. **b** RNA helicase proteins with significantly changed cysteines’ oxidation. Each bar represents oxidation level (% oxidation) of protein cysteine in fetal (red) and adult (blue) HSPCs. Columns displayed only for fetal HSPCs illustrate cysteines oxidized uniquely in fetal HSPCs. Positions of oxidized cysteines within the protein sequence and their localization within protein functional domain are indicated. Data represents mean ± SD in three biological replicates. **c** Overrepresented (p<0.05) protein domains of proteins with redox regulated cysteines in fetal and adult HSPCs classified by Interpro. **d** GO enrichment for proteins with cysteines significantly higher oxidized in adult HSPCs for overrepresentation of biological processes. Significantly enriched (p-value < 0.05) biological processes were selected and redundant GO terms were filtered out. Number of proteins within each process is shown to the right of each bar. **e** Proteins with significantly higher oxidized cysteines or proteins with cysteines oxidized only in fetal HSPCs being part of respiratory chain complexes. Each bar represents oxidation level (% oxidation) of protein cysteine in fetal (red) and adult (blue) HSPCs. Positions of oxidized cysteines within the protein sequence are indicated. Data represents mean ± SD in three biological replicates. **f** Heatmap represents oxidation level (%) of peptides with significantly changed cysteine oxidation (adjusted p-value < 0.05) and peptides with cysteines found to be oxidized only in fetus (3: 0 fetal: adult valid values; marked with red frames) that are involved in protein quality control in three biological replicates in fetal and adult HSPCs. **g** Schematic picture of effect of cysteine oxidation of heat shock protein HSP 90-alpha (Hsp90aa1) on ATPase activity. **h** Schematic picture of the effect of cysteine oxidation of Ubiquitin-like modifier-activating enzyme 1 (Uba1) on its function.

**Supplementary Figure 5. Polysome profiling of hESCs upon oxidative conditions. a** Heatmaps showing cysteine oxidation level (%) of peptides that belong to the proteins with significantly redox sensitive cysteines depicted in Fig. 5a in fetal and adult HSPCs that belong to the 43S PIC. Left heatmap depicts translation initiation factor subunits and right heatmap displays proteins of the small ribosomal subunit. Significantly changed peptides also displayed in Fig. 5a are marked as bold. Peptides with cysteines found to be oxidized only in fetus (3: 0 fetal: adult valid values) are marked with red frames). **b** Heatmap depicting average oxidation level (%) of all identified unique cysteine peptides belonging to eIF3b and eIF3d proteins detected in fetal and adult HSPCs and also in hESCs after treatment with 100 µM and 500 µM H_2_O_2_. n=5. **c** De novo protein synthesis measured by ^35^S radioactive methionine/cysteine incorporation in hESCs treated with 100 µM H_2_O_2_ (treated) or vehicle (Ctrl). Graph shows relative rate of protein synthesis ± SD in at least three independent experiments. *p-value < 0.05 (t-test). **d** Representative sucrose gradient profile of hESCs after incubation with 100 µM H_2_O_2_ (right) with indicated monosome and polysome fractions, and quantification of the heavy polysome fractions (12-14) (left). Bar chart represents mean ± SD from three biological replicates. **e** Gene set enrichment analysis (GSEA) on the normalized dataset of hESCs treated with 100 µM H_2_O_2_ (TREATED) and untreated cells (CTRL).

**Supplementary Figure 6. a** MLL-ENL expression following induction with doxycycline. **b** Volcano plot showing average log2 (MLL-ENL/WT) % oxidation for 2782 unique cysteine containing peptides plotted against −log10 adjusted p-value. Unique cysteine-containing peptides with significantly increased oxidation levels in fetal HSPCs upon MLL-ENL expression are shown in orange.

**Supplementary Figure 7. a** Experimental design of the iodoTMT (iTMT) labelling strategy in redox proteomic analysis of fetal (FL) and adult (ABM) HSPCs. **b** Experimental design of the iodoTMT (iTMT) labelling strategy in redox proteomic analysis of H9 human embryonic stem cells (hESC) in five experiments. **c** Experimental design of the TMT labelling strategy in immunoprecipitation experiment for H9 human embryonic stem cells (hESC).

**Supplementary Figure 8. Cysteine conservation among helicases and species. a** Sequences of identified RNA helicases were aligned and sorted by pairwise identity in Jalview. Redox sensitive cysteines that were conserved among other helicases with redox sensitive cysteines are indicated. **b** Sequences of RNA helicase with identified redox sensitive cysteine were aligned with orthologous sequences from selected species and sorted by pairwise identity in Jalview [2]. Redox sensitive cysteines that were conserved among species are indicated.

[1] Jassinskaja M*, et al.* Comprehensive Proteomic Characterization of Ontogenic Changes in Hematopoietic Stem and Progenitor Cells. *Cell Rep* **21**, 3285-3297 (2017).

[2] Waterhouse A.M, *et al.* Jalview Version 2—a multiple sequence alignment editor and analysis workbench, *Bioinformatics* **25**, Issue 9, 1 May 2009, Pages 1189–1191
